# Periplasmic chitooligosaccharide-binding protein requires a three-domain organization for substrate translocation

**DOI:** 10.1038/s41598-023-47253-y

**Published:** 2023-11-23

**Authors:** Takayuki Ohnuma, Jun Tsujii, Chikara Kataoka, Teruki Yoshimoto, Daijiro Takeshita, Outi Lampela, André H. Juffer, Wipa Suginta, Tamo Fukamizo

**Affiliations:** 1 Department of Advanced Bioscience, Kindai University, 3327-204 Nakamachi, Nara, 631-8505 Japan; 2 Agricultural Technology and Innovation Research Institute (ATIRI), Kindai University, 3327-204, Nakamachi, Nara, 631-8505 Japan; 3 Biomedical Research Institute, National Institute of Advanced Industrial Science and Technology (AIST), 1-1-1 Higashi, Tsukuba-Shi, Ibaraki, 305-8566 Japan; 4 Biocenter Oulu, University of Oulu, P.O. Box 5000, FI-90014 Oulu, Finland; 5 Faculty of Biochemistry and Molecular Medicine, University of Oulu, P.O.Box 5000, FI-90014 Oulu, Finland; 6 School of Biomolecular Science & Engineering, Vidyasirimedhi Institute of Science and Technology (VISTEC), Wangchan Valley 555 Moo 1 Payupnai, Wangchan, Rayong 21210 Thailand

**Keywords:** Proteins, Carbohydrates, Amino sugars, X-ray crystallography, Biochemistry, Structural biology, Molecular modelling

## Abstract

Periplasmic solute-binding proteins (SBPs) specific for chitooligosaccharides, (GlcNAc)_n_ (n = 2, 3, 4, 5 and 6), are involved in the uptake of chitinous nutrients and the negative control of chitin signal transduction in *Vibrios*. Most translocation processes by SBPs across the inner membrane have been explained thus far by two-domain open/closed mechanism. Here we propose three-domain mechanism of the (GlcNAc)_n_ translocation based on experiments using a recombinant *Vc*CBP, SBP specific for (GlcNAc)_n_ from *Vibrio cholerae*. X-ray crystal structures of unliganded or (GlcNAc)_3_-liganded *Vc*CBP solved at 1.2–1.6 Å revealed three distinct domains, the Upper1, Upper2 and Lower domains for this protein. Molecular dynamics simulation indicated that the motions of the three domains are independent and that in the (GlcNAc)_3_-liganded state the Upper2/Lower interface fluctuated more intensively, compared to the Upper1/Lower interface. The Upper1/Lower interface bound two GlcNAc residues tightly, while the Upper2/Lower interface appeared to loosen and release the bound sugar molecule. The three-domain mechanism proposed here was fully supported by binding data obtained by thermal unfolding experiments and ITC, and may be applicable to other translocation systems involving SBPs belonging to the same cluster.

## Introduction

Chitin is a β-1,4-linked polysaccharide of *N*-acetylglucosamine (GlcNAc), and is degraded by chitinases^1^, β-*N*-acetylglucosaminidases^2^, chitin deacetylases^3^ and lytic polysaccharide monooxygenases^4^, to produce GlcNAc, chitin oligosaccharides, (GlcNAc)_n_ (n = 2, 3, 4, 5, and 6) and their de-*N*-acetylated derivatives. The products are utilized as structural components in various chitin-containing organisms^5^ or as carbon and nitrogen sources by chitin consumers^6^. *Vibrio* spp. live in the marine ecosystem and possess all these enzymes, enabling them to efficiently catabolize chitin as their nutritional source^7^. The enzymatic degradation of chitin by extracellular chitinases in *Vibrio*s produces (GlcNAc)_n_^8–10^, which are taken up into the periplasm through a chitoporin localized in the outer membrane^11–14^. (GlcNAc)_n_ transported into the periplasm undergo further enzymatic degradation^15–17^ and are trapped by a periplasmic solute-binding protein (SBP) specific for (GlcNAc)_n_^18^. Various SBPs have been recognized to mediate the transport of the corresponding solutes into the cytoplasm through a specific transporter localized in the inner membrane^19^; furthermore, they interact with signal receptor proteins to control the signal response^20^. SBPs specific to (GlcNAc)_n_ (referred to as CBP) also have such a dual functionality; (GlcNAc)_n_-liganded or unliganded CBPs interact with a chitin sensing protein as well as a (GlcNAc)_2_-specific ABC-transporter^21,22^. A large conformational change of CBP from “open” to “closed”, or vice versa, induced by binding/release of (GlcNAc)_n_^19^ triggers a series of interactions involving sensing or transporter proteins. It is therefore highly desirable to thoroughly analyze the structure, binding mechanism of CBP/(GlcNAc)_n_ and the CBP/(GlcNAc)_n_/membrane protein interactions.

**Figure 1:**
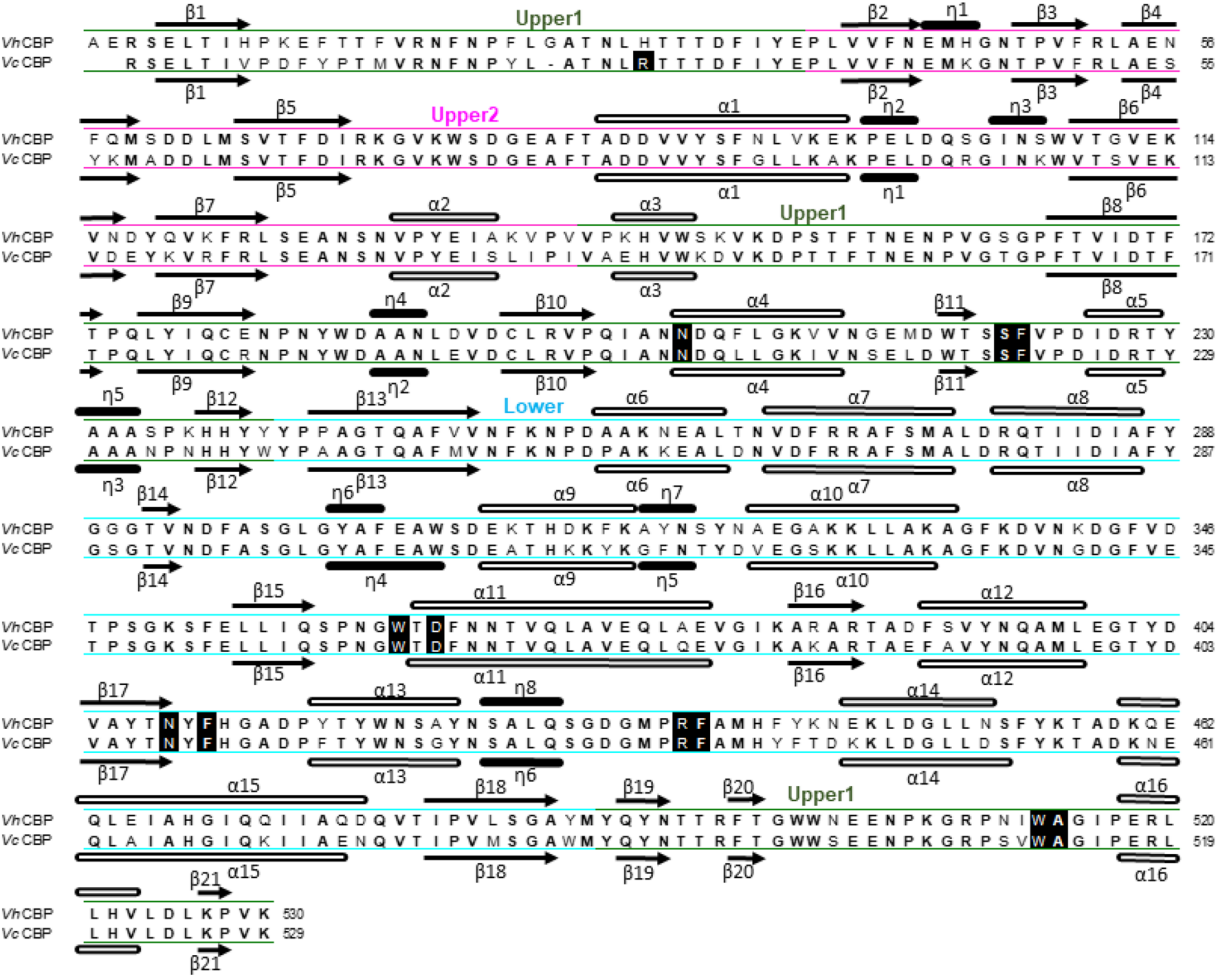
Sequence alignment of the periplasmic solute-binding proteins specific to (GlcNAc)_2_ from *Vibrios*. Identical amino acids are written in bold. The secondary structures are designated α1, α2, α3, … (α-helices, open bars), β1, β2, β3,… (β-strands, arrows), and η1, η2, η3, … (3_10_-helices, filled bars) from the N-terminus. The sequence of Upper1 domain is underlined (*Vc*CBP) or overlined (*Vh*CBP) in dark green, Upper2 domain in magenta, and Lower domain in cyan. Amino acid residues, which interact with the ligand and are conserved between *Vh*CBP and *Vc*CBP, are highlighted on a black background.

Sheepers et al.^23^ classified SBPs into seven clusters, A, B, C, D, E, F and G, according to their crystal structures, and cluster C was subdivided into five subclusters (I, II, III, IV and V) in our previous review^24^. Two CBPs from *Vibrios* have been investigated so far. One is from *V. cholerae* (*Vc*CBP)*,* a facultative anaerobe that causes a deadly disease to humans called cholerae^25^, and the second is from *V. campbelli*, (formerly *V. harveyi*, *Vh*CBP), a bioluminescence marine bacterium that causes a fatal disease to aquatic animal called Vibriosis^26^. Both *Vc*CBP and *Vh*CBP belong to cluster C/subcluster IV (C-IV), in which SBPs are specific for oligosaccharides, including (GlcNAc)_n_, mannooligosaccharides^27^, and cellooligosaccharides^28^. The first crystal structures of CBP were solved for *Vc*CBP and deposited in the PDB database in 2006; PDB codes 1ZTY for unliganded (open) *Vc*CBP and 1ZU0 for (GlcNAc)_2_-liganded (closed) *Vc*CBP, and in 2013; PDB codes, 4GF8 for unliganded (open) *Vc*CBP and 4GFR for (GlcNAc)_2_-liganded (closed) *Vc*CBP. However, no functional or mechanistic information on this CBP was reported. Suginta et al.^29^ solved the crystal structure of a closed form of (GlcNA)_2_-liganded *Vh*CBP, with an amino acid sequence that shares 83% identity with that of *Vc*CBP as shown in Fig. 1. They regarded this protein as comprising two domains (the Upper and Lower domains) connected by a flexible hinge region, which enabled CBP to embrace (GlcNAc)_n_ in its substrate-binding groove lying between the two domains. Conserved Asp365, Phe437, Trp363 and Trp513 (Fig. 1) localized in the binding groove were found to contribute strongly to the interaction with (GlcNAc)_2_^30^. Most other studies on SBPs proposed that the proteins adopt a two-domain conformation, which is converted from the open state to the closed state upon capture of their substrates^31–33^. Kitaoku et al.^30^ found a suggestive conformation, “half-open” form, in the crystal structure of mutant *Vh*CBP (W513A), which provided some clues for the elucidation of the mechanism of (GlcNAc)_n_ translocation by CBP. Moreover, a three-domain organization was proposed for the SBPs belonging to cluster C and referred to as domains I, II, and III^19^. Further studies are needed to gain more extensive insights into the domain organization of CBPs.

In this study, we conducted in-depth investigations of the structure and mechanism of CBP by means of crystallography, molecular dynamics simulation, thermal unfolding experiments and isothermal titration calorimetry (ITC), using *Vc*CBP and (GlcNAc)_n_ (n = 2, 3, 4, 5 and 6). Based on the crystal structures and molecular dynamics simulation, we here propose that the three domains, Upper1, Upper2, and Lower domains, which correspond to domains I, II, and III, respectively^19^, are required to explain the translocation process of (GlcNAc)_2_ by CBP. This proposal was well supported by the thermodynamic data for (GlcNAc)_n_ binding to *Vc*CBP.

## Results

### Crystal structures of *Vc*CBP

**Figure 2:**
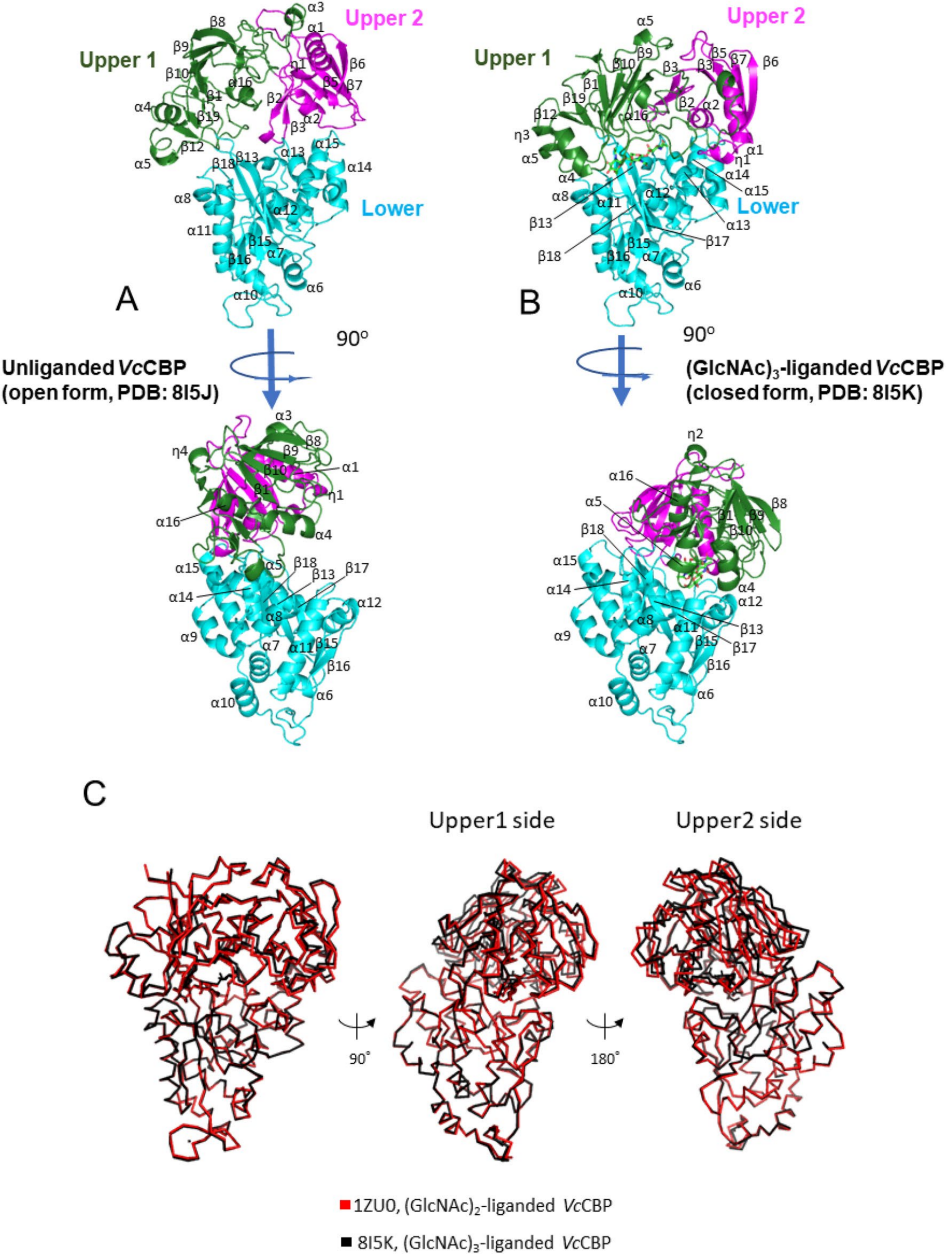
Crystal structures of unliganded *Vc*CBP (A, open state; 8I5J) and (GlcNAc)_3_-liganded *Vc*CBP (B, closed state; 8I5K). The upper panels represent views from the front, and the lower panels show side views. Upper1 domain is colored dark green, Upper2 magenta, and Lower cyan. Bound (GlcNAc)_3_ is represented by stick model colored light green. The secondary structures are designated as α1, α2, α3,…, β1, β2, β3,…, and η1, η2, η3,…, from the N-terminus, and correspond to those shown in Fig. 1. (**C**) Superimposition of the structures of (GlcNAc)_3_-liganded (8I5K, black) and (GlcNAc)_2_-liganded *Vc*CBP (1ZU0, red). Left, view from the front of the binding groove; middle, view from the Upper1 side; right, view from the Upper2 side.

We solved the structure of unliganded protein (open form) at 1.6 Å (Fig. 2A), a higher resolution than the *Vc*CBP structures formerly deposited (2.2–2.3 Å resolution). The closed structure of (GlcNAc)_3_-liganded *Vc*CBP was also solved at 1.22 Å resolution (Fig. 2B). The coordinates were deposited in the database with the PDB codes 8I5J and 8I5K, respectively. The statistics for the crystallization and refinement procedures are listed in Table 1. Superimposition of the unliganded structure (open form) obtained here with that of 1ZTY previously deposited revealed that these two structures are almost identical, with a root-mean-square deviation (RMSD) of 0.600 Å, although the space group of 8I5J (*P*2_1_) was different from that of 1ZTY (*P*3_2_21). The RMSD values obtained from the superimpositions of (GlcNAc)_3_-liganded structure (8I5K) with those of (GlcNAc)_2_-liganded *Vc*CBP (1ZU0) and (GlcNAc)_3_-liganded *Vh*CBP (6LZQ^30^) were 0.529 Å and 0.393 Å, respectively. Both structures obtained here for *Vc*CBP showed three distinct domains, the Upper1, Upper2 and Lower domains (Fig. 2A,B). Compared with the unliganded structure, the angle of closure between Upper1 and Lower domains of the (GlcNAc)_3_-liganded structure was found to be 54.7°. This was determined from the rotation angle of the Gln490 α-carbon atom (Upper1 domain) using the Ala486 α-carbon, which is located at the hinge, as the fixed rotation center. The Upper1 region is colored in deep green, Upper2 magenta, and Lower cyan. Upper1 (1–35, 140–239, and 489-C-terminal) comprises α3-α5/α16, β1/β8-β12/β19-β21 and η2–η3, while Upper2 (36–139) comprises α1-α2, β2-β7 and η1. The Lower domain (240–488) comprises α6-α15, β13-β18 and η4–η6 (Figs. 1, 2A,B). The division of the upper structural region into Upper1 and Upper2 domains appeared to be reasonable from the superimposition of (GlcNAc)_2_-liganded (1ZU0) and (GlcNAc)_3_-liganded *Vc*CBP (8I5K) (Fig. 2C), where the Lower domain fully overlapped (RMSD, 0.306 Å), while the Upper1 domain partly deviated (RMSD, 1.203 Å) and the Upper2 domain strongly deviated (RMSD, 1.930 Å). The structure liganded with (GlcNAc)_3_ differed slightly from that with (GlcNAc)_2_ especially in the Upper2 domain. Accommodating the reducing-end GlcNAc of the bound (GlcNAc)_3_ appeared to affect more strongly the Upper2 domain.

**Table 1:** Data-collection and refinement statistics.

	Unliganded *Vc*CBP	(GlcNAc)_3_-liganded *Vc*CBP
Data collection		
Wavelength (Å)	0.9800	0.9800
Space group	*P*2_1_	*C*2
Unit-cell parameters		
*a*, *b*, *c* (Å)	*a* = 81.12, *b* = 81.64, *c* = 87.27	*a* = 135.09, *b* = 51.68, *c* = 70.33
*α*, *β*, *γ* (°)	*α* = 90, *β* = 104.15, *γ* = 90	*α* = 90, *β* = 98.83, *γ* = 90
Resolution (Å)	20.0–1.60	20.0–1.22
	(1.69–1.60)	(1.28–1.22)
Completeness (%)	91.6 (61.7)	84.8 (41.0)
*R*_sym_	0.097 (0.763)	0.079 (0.771)
*I*/σ (*I*)	8.75 (1.56)	8.82 (1.29)
Redundancy	3.3 (2.6)	3.4 (2.8)
*CC*_1/2_	0.994 (0.519)	0.996 (0.507)
Refinement		
Resolution (Å)	19.8–1.60	19.3–1.22
No. of reflections	261,323	237,863
* R*_work_/*R*_free_ (%)	16.2/19.1	16.9/18.7
No. of atoms		
Protein	8475	4244
Ligand		44
Ion	2	1
Water	773	563
* B*-factors (Å^2^)		
Protein	22.6	17.4
Ligand		13.0
Ion	25.5	14.9
Water	34.2	28.5
R.m.s.d’s		
Bond lengths (Å)	0.009	0.007
Bond angles (°)	0.97	0.94
Ramachandran plot		
Favored region (%)	96.7	97.3
Allowed region (%)	3.3	2.7
Disallowed region (%)	0	0

Values in parentheses are for the highest resolution shell.

### Binding mode of (GlcNAc)_3_

**Figure 3:**
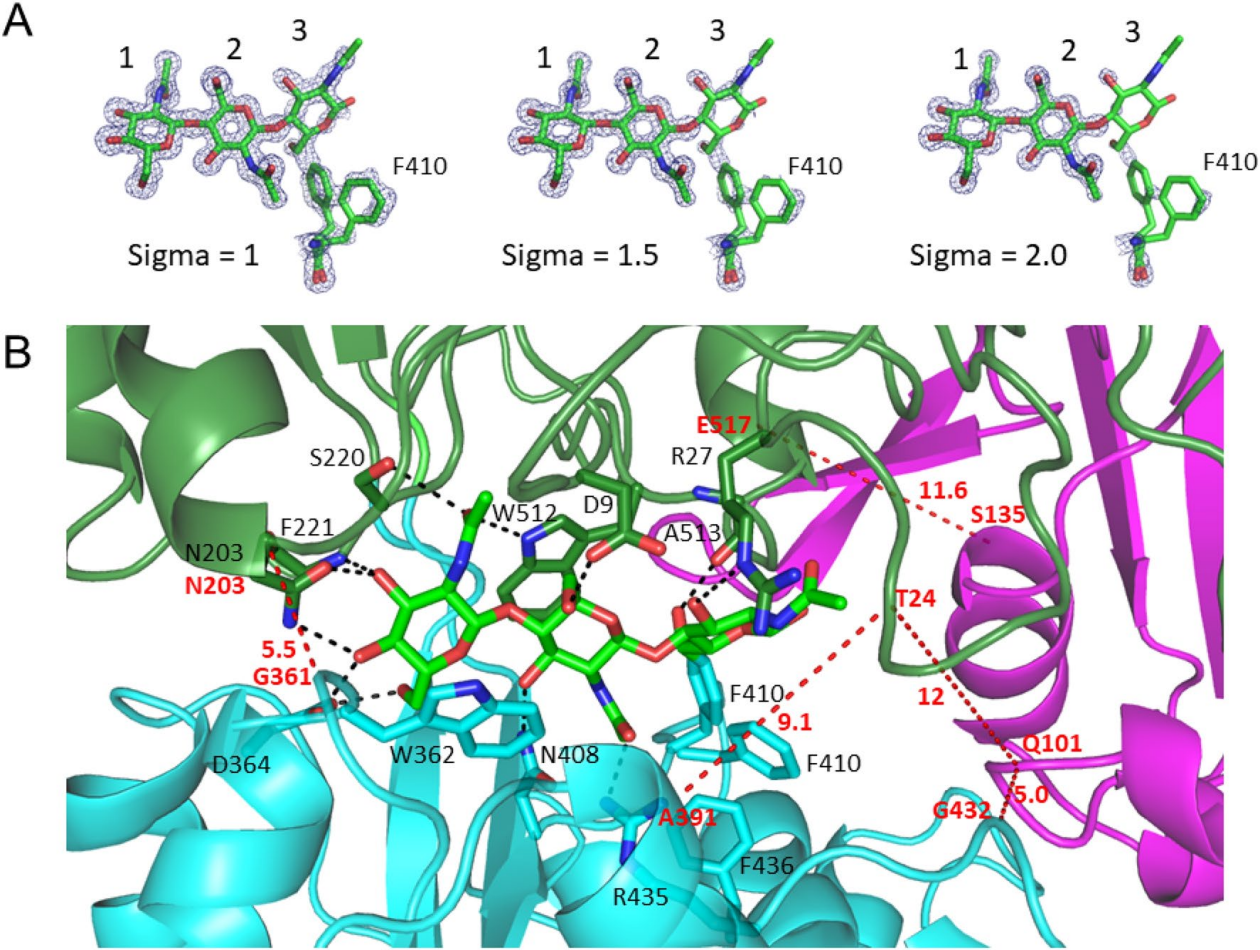
State of (GlcNAc)_3_ bound to *Vc*CBP. (**A**) 2*F*_0_-*F*_c_ map of the bound (GlcNAc)_3_ and the Phe410 side chain. The occupancies of the Site3 GlcNAc and two conformers of the Phe410 side chain were each assumed to be 0.5. (**B**) Binding mode. The color system is identical to that in Fig. 2. The possible hydrogen bonds are shown by black broken lines. The red broken lines represent the distances between the α-carbons monitored in the molecular dynamics simulation shown in Fig. 6.

Figure 3A shows a 2*F*_0_–*F*c map of the bound (GlcNAc)_3_. As in the case of *Vh*CBP^29^, electron density of (GlcNAc)_3_ was identified in the (GlcNAc)_3_-liganded *Vc*CBP. Since Kitaoku et al.^30^ designated these subsites as Site1, Site2 and Site3, the same subsite nomenclature was used in this study. In the (GlcNAc)_3_-liganded *Vc*CBP, the electron density of the Site3 GlcNAc was lower than those of the two GlcNAc residues at Site1 and Site2, and the occupancy of Site3 GlcNAc was set at 0.5. Two conformers of the Phe410 side chain were observed near the Site3 GlcNAc, and the individual occupancies of the side chain were also set at 0.5. It is most likely that the mobility of the Site3 GlcNAc is much higher than that of the other GlcNAc residues and that the Phe410 side chain flips alternatively back and forth in the liganded structure. Both GlcNAc units at Site1 and Site2 appeared to be hydrogen-bonded or in hydrophobic contact with Asp9, Asn203, Ser220, Phe221, Trp362, Asp364, Asn408, Phe410, Arg435, Phe436 and Trp512, of which all except Asp9 are conserved between *Vh*CBP and *Vc*CBP (highlighted on a black background in Figs. 1 and 3B), while the GlcNAc unit at Site3 makes only a couple of hydrogen bonds with the main chain carbonyl of Ala513 and the guanidyl nitrogen of Arg27, which are localized to the interface between the Upper1 and Upper2 domains (Fig. 3B, Supplementary Table S1).

**Figure 4:**
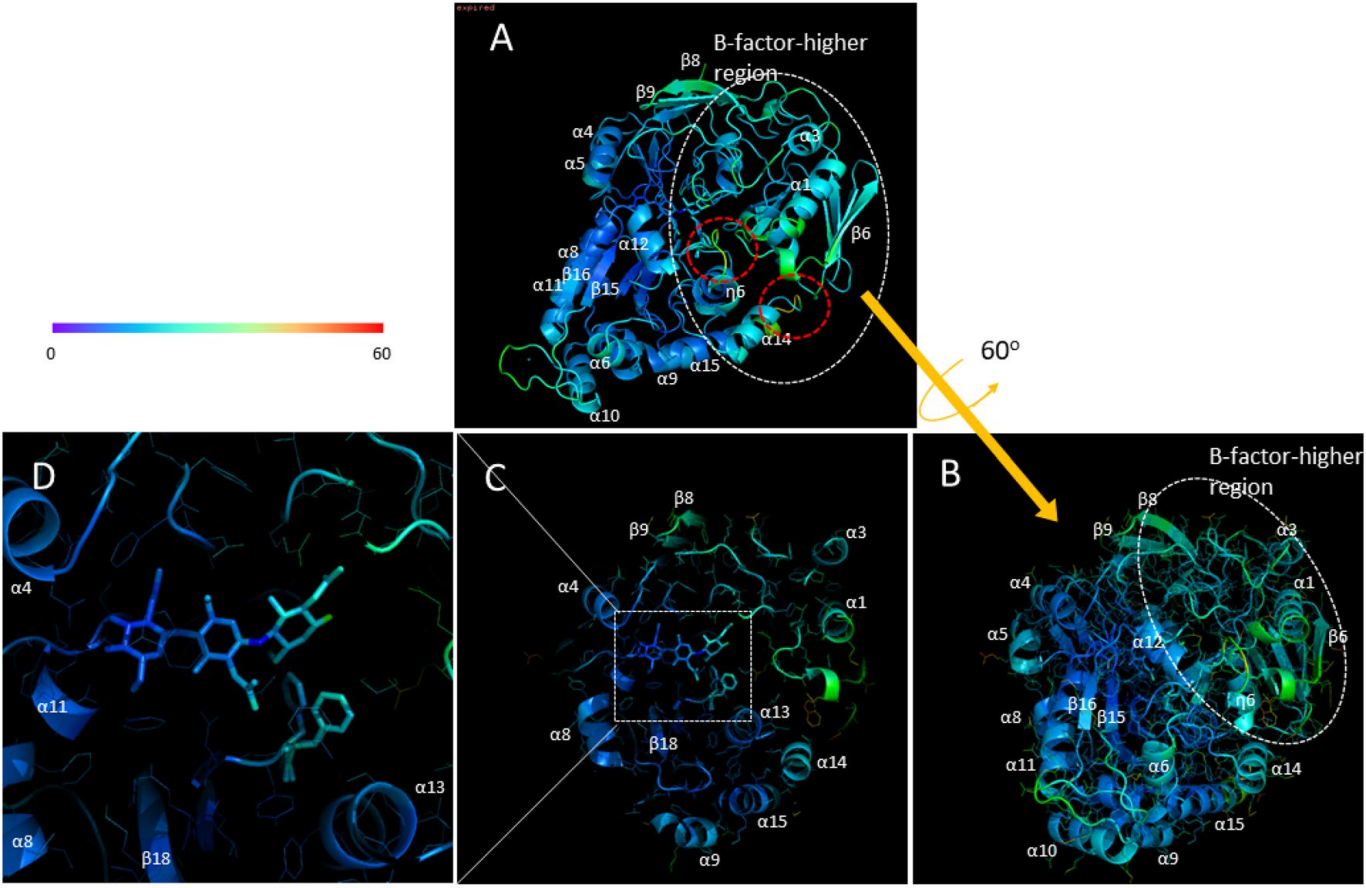
Visualization of crystallographic B-factors for the *Vc*CBP structure. (**A**) and (**B**) entire *Vc*CBP structure in complex with (GlcNAc)_3_; (**C**) Cross-section of the (GlcNAc)_3_-binding site; and (**D**), close-up view of the (GlcNAc)_3_-binding site. The colors from violet to blue, green, yellow, orange and red indicate B factors from small to large. Broken circles colored white represent B-factor-higher regions, while those colored red represent loop regions with the highest B-factors.

B-factor values visualized in the main chain structure of (GlcNAc)_3_-liganded *Vc*CBP are shown in Fig. 4A, and were higher in the Upper2/Lower interface as well as the Site3-GlcNAc contact surface of Upper1 domain. Notably, the B-factors were significantly higher at the loop region immediately following the secondary structure η6 and the loop between α14 and α15 (red broken circles, Fig. 4A). These two loops are located at the Upper2-Lower interface. Furthermore, B-factor values of the individual GlcNAc residues were 9.86, 12.67 and 16.28 Å^2^, respectively (Fig. 4D), indicating the higher mobility of the Site3 GlcNAc. These B-factor data were fully consistent with the electron density data described above. The cooperative motions of the Site3 GlcNAc and the Phe410 side chain may be significant from the mechanistic viewpoint.

### Molecular dynamics simulation

**Figure 5:**
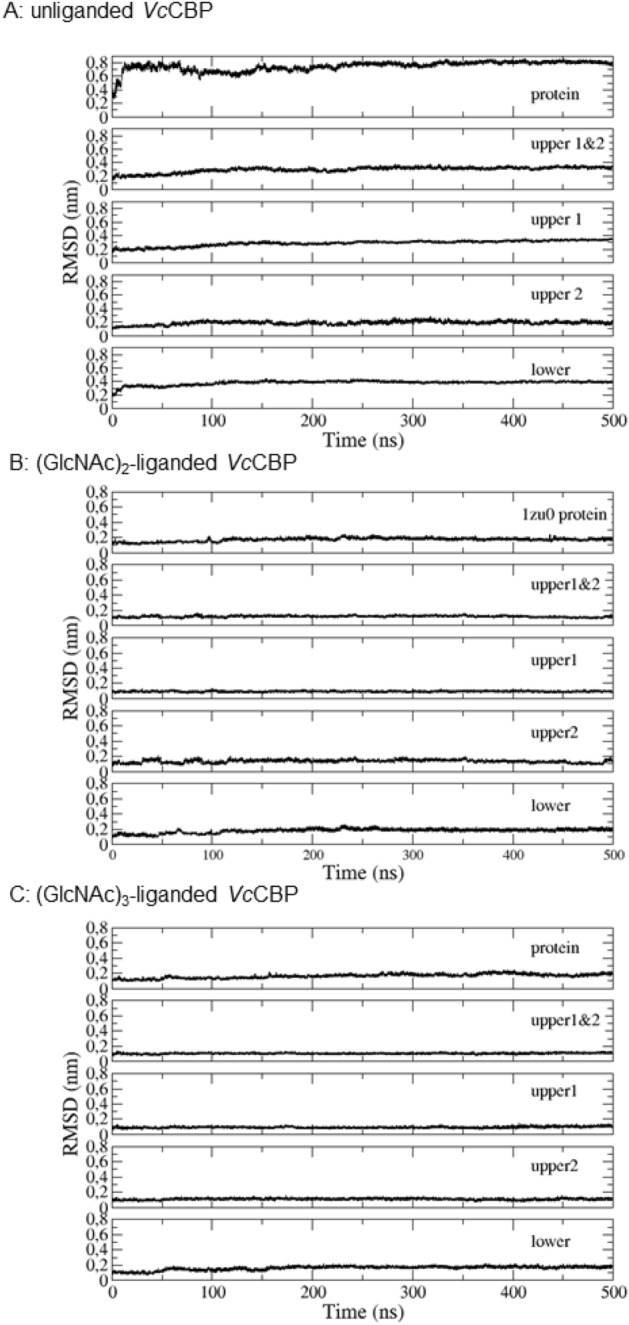
Time-dependent backbone RMSDs calculated by molecular dynamics simulation. Unliganded *Vc*CBP (**A**, 8I5J), (GlcNAc)_2_-liganded *Vc*CBP (**B**, 1ZU0) and (GlcNAc)_3_-liganded *Vc*CBP (**C**, 8I5K). From top to bottom: the entire protein structure, Upper1 + Upper2, Upper1, Upper2, and Lower domains.

Figure 5 shows the time-dependent RMSD for unliganded, (GlcNAc)_2_-liganded and (GlcNAc)_3_-liganded *Vc*CBP. The unliganded form exhibited the largest motion (RMSD, 0.8 nm; the top panel of Fig. 5A) of the entire protein molecule, while the motions of the individual domains remained at lower levels (RMSD, 0.1–0.3 nm; Fig. 5A). This clearly indicated a domain motion, in which the individual domains did not change their own conformations but changed their relative arrangement. On the other hand, the motions of the entire protein molecules as well as individual domains were very low in the (GlcNAc)_2_-liganded and (GlcNAc)_3_-liganded *Vc*CBP, where RMSDs were only 0.1–0.2 nm (Fig. 5B,C).

**Figure 6:**
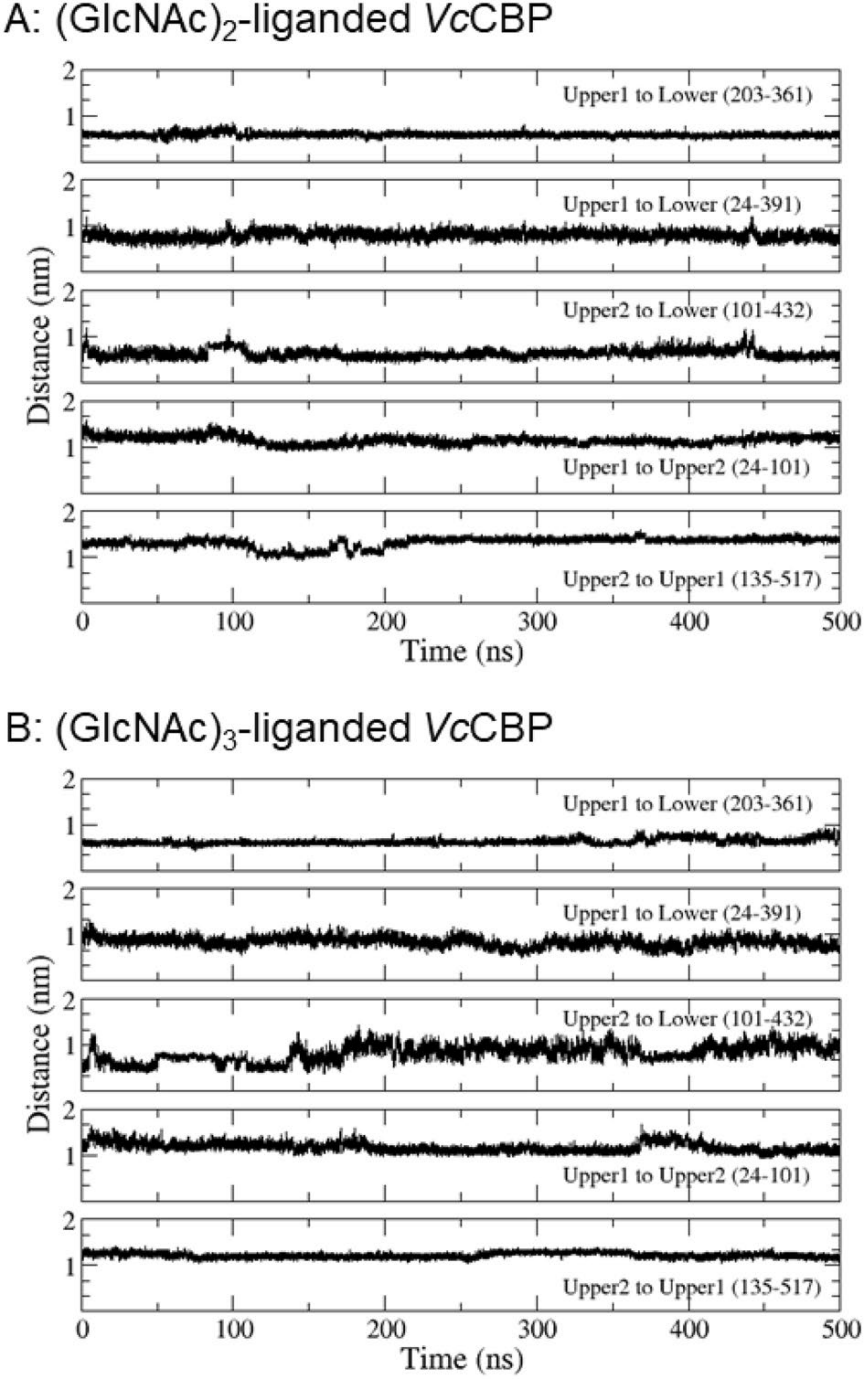
The selected, time-dependent interatomic distances between α-carbons of the amino acids. From top to bottom: Asn203-Gly361 (Upper1-Lower), Thr24-Ala391 (Upper1-Lower), Gln101-Gly432 (Upper2-Lower), Thr24-Gln101 (Upper1-Upper2) and Ser135-Glu517 (Upper2-Upper1), for (GlcNAc)_2_-liganded *Vc*CBP (**A**, 1ZU0) and (GlcNAc)_3_-liganded *Vc*CBP (**B**, 8I5K).

**Figure 7:**
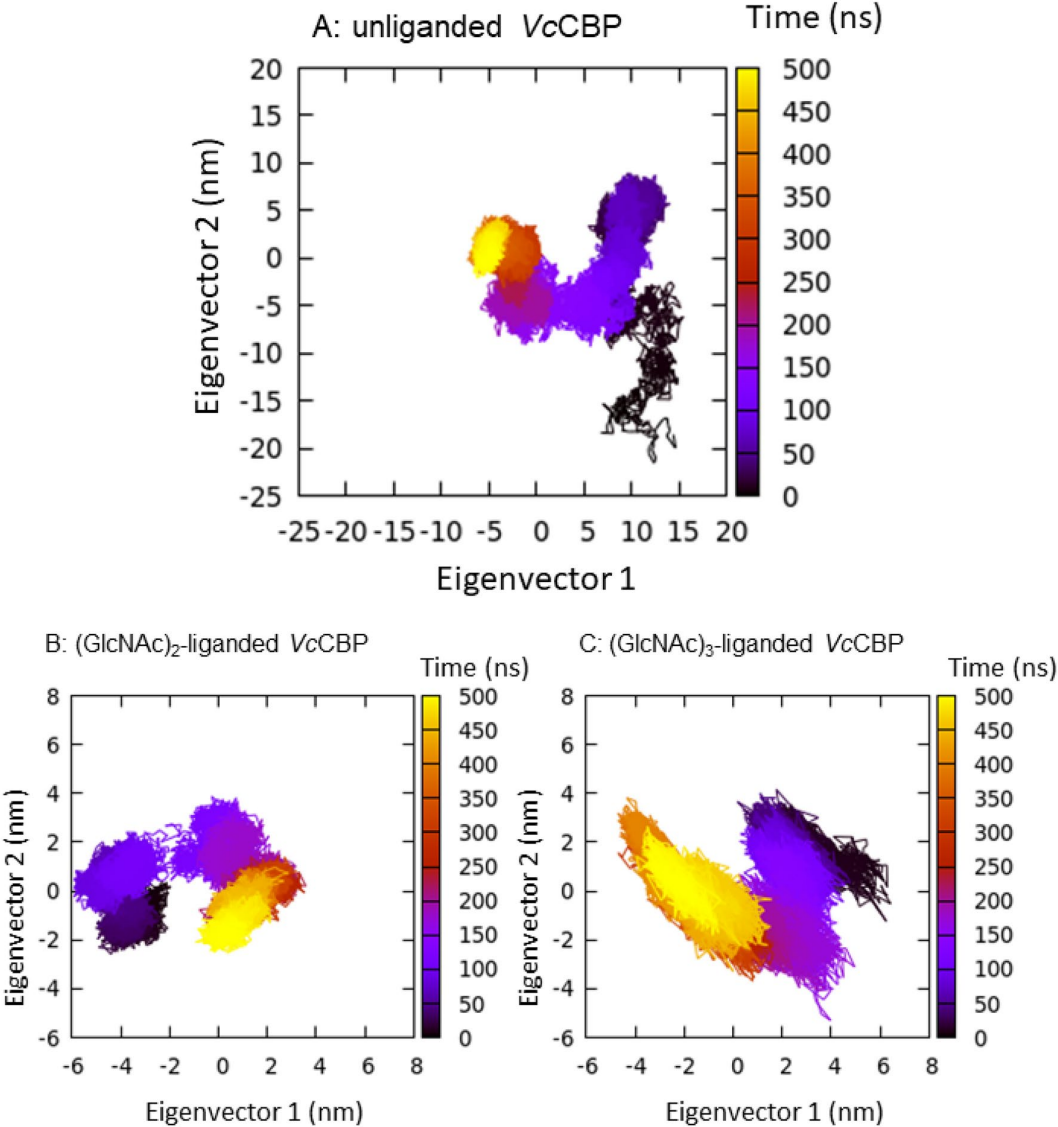
Two-dimensional projections of eigenvector1 and eigenvector2 showing the time-dependent conformational changes of unliganded (**A**), (GlcNAc)_2_-liganded (**B**) and (GlcNAc)_3_-liganded *Vc*CBP (**C**). The color changes from black, violet, purple, magenta, red, orange and to yellow with progress of the simulation time.

Figure 6 shows the distances between the α-carbons of the two numbered amino acid residues (amino acids labeled in red, Fig. 3B). The distance 203–361, which reflects the separation between Upper1 and Lower domains (red broken line between Asn203 and Gly361 in Fig. 3B; the top panels of Fig. 6A,B), did not significantly differ between (GlcNAc)_2_-liganded and (GlcNAc)_3_-liganded *Vc*CBP. For the distance 24–391 (red broken line between Thr24 and Ala391 in Fig. 3B; second panels from the top of Fig. 6A,B), which also reflects the separation between Upper1 and Lower domains at another site, no significant difference was observed between (GlcNAc)_2_-liganded and (GlcNAc)_3_-liganded *Vc*CBP. Furthermore, the distance 135–517 (red broken line between Ser135 and Glu517 in Fig. 3B; the bottom panels of Fig. 6A,B) reflecting the separation between the Upper1 and Upper2 domains, did not exhibit any differences between the two liganded *Vc*CBP. However, the distances 101–432 (Gln101–Gly432) and 24–101 (Thr24-Gln101) reflecting the separations of Upper2-Lower and Upper1–Upper2, respectively, fluctuated more in (GlcNAc)_3_-liganded *Vc*CBP than in (GlcNAc)_2_-liganded (the third and fourth panels from the top of Fig. 6A,B). The movements are more intensive in the (GlcNAc)_3_-liganded *Vc*CBP than in the (GlcNAc)_2_-liganded in this region. Elongation of the chain of the bound oligosaccharide from (GlcNAc)_2_ to (GlcNAc)_3_ was found to enhance the molecular motion around amino acid residue Gln101 located in the Upper2 domain. The larger conformational change in (GlcNAc)_3_-liganded *Vc*CBP was also confirmed from Fig. 7, which shows the 2-dimensional projection of the trajectories of principal components, eigenvector1 and eigenvector2. In unliganded *Vc*CBP, essential subspaces were widely extended as compared with the liganded *Vc*BP (Fig. 7A; eigenvector1/eigenvector2, -7 ~ 15 nm/-22 ~ 9 nm) revealing a large conformational change from the open state to the closed state (Fig. 2A,B). Although the conformational spaces of the liganded states were much less extensive (Fig. 7B; − 6 ~ 3.5 nm/− 2.5 ~ 3.5 nm and Fig. 7C; − 4.5 ~ 6.3 nm/− 5.5 ~ 4.0 nm), (GlcNAc)_3_-liganded *Vc*CBP was found to occupy a larger conformational space than (GlcNAc)_2_-liganded, indicating that the structural variations induced by (GlcNAc)_3_ binding to Site1, Site2 and Site3 were more abundant than those induced by (GlcNAc)_2_ binding to Site1 and Site2.

**Figure 8:**
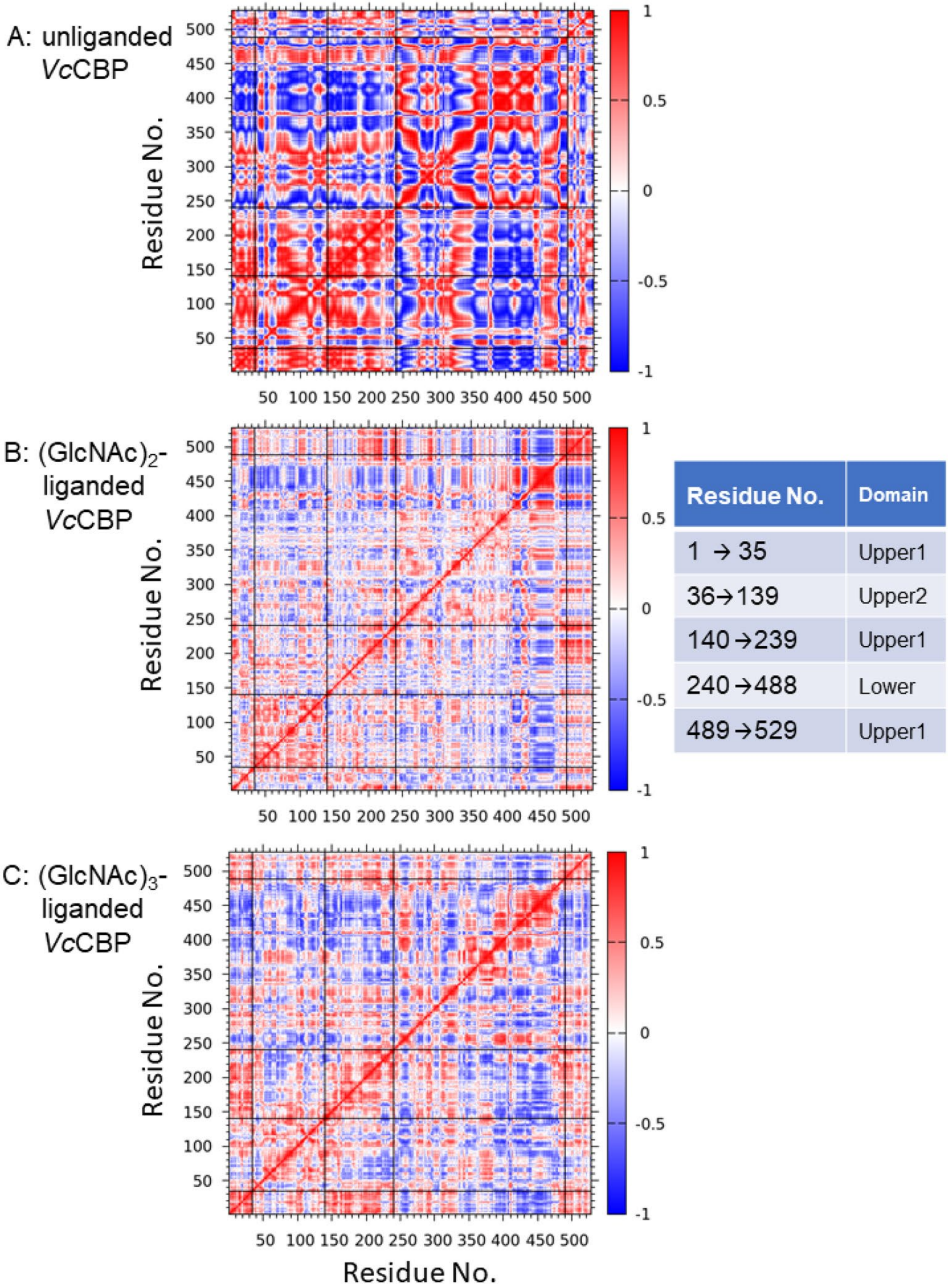
Cross correlation heat maps of Cα atoms around their mean positions for the entire simulation period. (**A**) Unliganded *Vc*CBP (8I5J); (**B**) (GlcNAc)_2_-liganded *Vc*CBP (1ZU0); and (**C**) (GlcNAc)_3_-liganded *Vc*CBP (8I5K). The color gradation from red to blue corresponds to extents of correlated motions (from 1 to 0, positive correlations) and anti-correlated motions (from 0 to − 1, negative correlations). The horizontal and vertical axes represent the amino acid residue No. of *Vc*CBP. The thin guided lines in the figures represent the boundaries of the individual domains, Upper1, Upper2, and Lower. The correspondence table on the right shows which domain is located where.

A cross-correlation heat map was generated based on the Cα-atom positions using the ProDy python package. The results are shown in Fig. 8. In unliganded *Vc*CBP (Fig. 8A), strong self-positive correlations (red) were found in the region of residue No. 1-239, which corresponds to Upper1 + Upper2 domains, and also in residue No. 350-440, which includes amino acid residues interacting with the ligand from Lower domain (Fig. 3B and Supplementary Table S1). However, strong inter-negative correlations (blue) were found between 1 and 239 (Upper1 + Upper2) and 350–440 (the interacting region of Lower domain). This clearly indicated a large domain motion from open to closed, vice versa, shown in Fig. 2A and B. Although the correlations were weaker in (GlcNAc)_3_-liganded *Vc*CBP (Fig. 8C) than in the unliganded state, we observed self-positive correlations (red) in residue No. 36-139 (Upper2 domain); however, the inter-correlations between 36 and 139 (Upper2) and 140–239 (inner Upper1) and between 36 and 139 (Upper2) and 1–35 (N-terminal Upper1) were rather negative (blue) as compared with the unliganded state. Furthermore, inter-correlations between 140 and 239 (inner Upper1) and 1–35 (N-terminal Upper1) were rather positive (red). It was clear that Upper1 and Upper2 domains move unitedly in the unliganded state, but that these two domains move independently in the (GlcNAc)_3_-liganded state. For the (GlcNAc)_2_-liganded *Vc*CBP (Fig. 8B), although correlations were even weaker than (GlcNAc)_3_-liganded state, similar trends were observed in the region corresponding to 1–239 (Upper1 + Upper2). Weak but significant inter-negative correlations (blue) between Upper1 + Uppe2 (1–239) and the ligand-interacting region of Lower domain (350–470) were also found in the two liganded states (Fig. 8B,C). However, the negative correlations were more intensive in the (GlcNAc)_3_-liganded state than in the (GlcNAc)_2_-liganded, indicating that the movements were enhanced in (GlcNAc)_3_-liganded *Vc*CBP (Fig. 8C) as compared with (GlcNAc)_2_-liganded state (Fig. 8B).

### Thermal unfolding experiments with ***Vc***CBP in the presence of (GlcNAc)_n_

A typical dataset of the thermal unfolding transitions of *Vc*CBP in the absence or presence of (GlcNAc)_n_ was shown in Supplementary Fig. S1. The unfolding transition was highly cooperative, with the fraction unfolded increasing sharply at the transition temperature (*T*_m_). The individual unfolding experiments were conducted twice, and the averaged values of *T*_m_ were calculated as listed in Table 2. Thermal unfolding of unliganded *Vc*CBP took place at *T*_m_ of 43.2 °C. When (GlcNAc)_2_, (GlcNAc)_3_ or (GlcNAc)_4_ was added to *Vc*CBP, *T*_m_ was elevated from 43.2 to 54.4 °C (Δ*T*_m_ = 11.2 °C), 54.8 °C (Δ*T*_m_ = 11.6 °C) and 53.8 °C (Δ*T*_m_ = 10.6 °C), respectively. As in the case of *Vh*CBP^29^, these three oligosaccharides bound strongly to *Vc*CBP. However, Δ*T*_m_ values induced by the addition of (GlcNAc)_5_ and (GlcNAc)_6_ were moderate, 6.3 °C for both, indicating that these two oligosaccharides bind to *Vc*CBP with significantly lower affinities^34^. Δ*T*_m_ for GlcNAc was only 2.8 °C, suggesting that the monomer bound to *Vc*CBP with much lower affinity.

**Table 2:** Transition temperatures of thermal unfolding of *Vc*CBP in the presence of GlcNAc and (GlcNAc)_n_ (n = 2, 3, 4, 5, or 6).

	*T*_m_ (°C)	Δ*T*_m_ (°C)
*Vc*CBP alone	43.2 ± 1.2	
+ GlcNAc	46.0 ± 1.0	2.8
+ (GlcNAc)_2_	54.4 ± 0.1	11.2
+ (GlcNAc)_3_	54.8 ± 0.3	11.6
+ (GlcNAc)_4_	53.8 ± 0.0	10.6
+ (GlcNAc)_5_	49.6 ± 0.3	6.3
+ (GlcNAc)_6_	49.6 ± 0.2	6.3

The unfolding experiments were conducted in 20 mM Tris–HCl buffer pH 8.0, monitoring the CD value at 222 nm using a Jasco J-720 spectropolarimeter (0.1 cm of cell length). Final concentrations of the protein and (GlcNAc)_n_ were 8 µM and 8 mM, respectively. Two repeated experiments were conducted to obtain the individual *T*_m_ values.

### Thermodynamic parameters for binding of (GlcNAc)_n_ (n = 2, 3, and 4)

**Figure 9:**
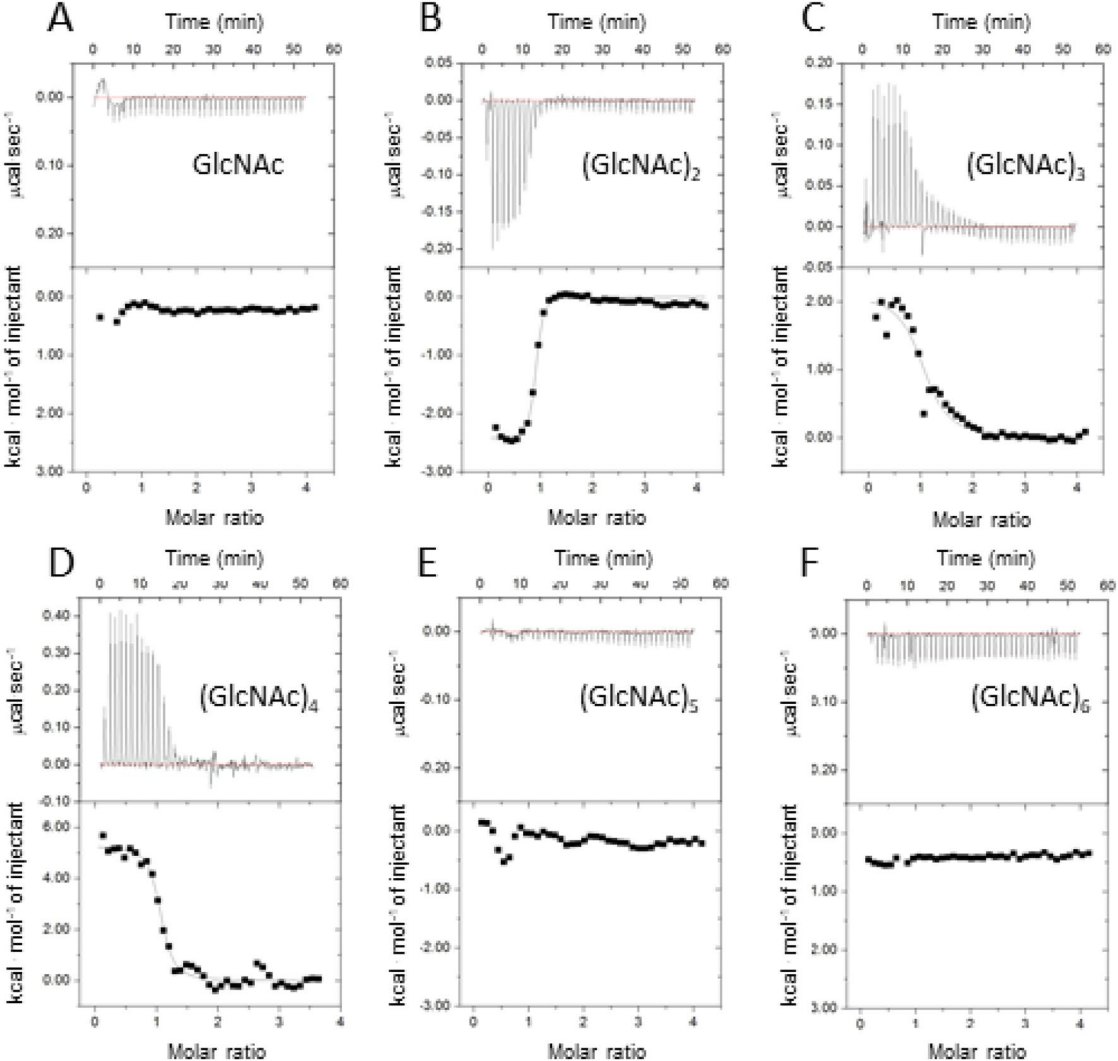
Thermograms and the theoretical fits obtained by ITC analysis. Titrations of *Vc*CBP with GlcNAc (**A**), (GlcNAc)_2_ (**B**), (GlcNAc)_3_ (**C**), (GlcNAc)_4_ (**D**), (GlcNAc)_5_ (**E**) and (GlcNAc)_6_ (**F**), were conducted in 20 mM Tris–HCl buffer, pH 8.0 at 25 °C. An iTC200 system (MicroCal Co.) was used to collect and analyze the experimental data. Other reaction conditions are described in the “Methods” section.

ITC experiments were conducted at 25 °C by titrating a 1.0 mM solution of GlcNAc or (GlcNAc)_n_ (n = 2, 3, 4, 5 or 6) into *Vc*CBP solution (50 µM). As shown in Fig. 9A–F, titrations with GlcNAc, (GlcNAc)_5_, or (GlcNAc)_6_ did not result in any heat release/absorption, whereas (GlcNAc)_2_, (GlcNAc)_3_ and (GlcNAc)_4_ exhibited clear heat release or absorption. This was consistent with the experimental data obtained from thermal unfolding experiments (Supplementary Fig. S1 and Table 2). It is noteworthy that titration with (GlcNAc)_2_ resulted in heat release but (GlcNAc)_3_ and (GlcNAc)_4_ resulted in absorption. The thermodynamic mechanism of the interaction with (GlcNAc)_2_ appears to be basically different from those with (GlcNAc)_3_ and (GlcNAc)_4_. Thermodynamic parameters were obtained based on the experimental data as listed in Table 3. For (GlcNAc)_2_, the enthalpic contribution (Δ*H*°) and the association constant (*K*_assoc_) were obtained based on the theoretical fit and were − 2.40 kcal mol^−1^ and 4.08 × 10^6^ M^−1^, respectively. Thus the binding free energy (Δ*G*°) was − 8.85 kcal mol^−1^. The entropic contribution (*− T*Δ*S*°) was calculated to be − 6.45 kcal mol^−1^. The interaction was entropy-driven with a smaller enthalpy gain. When (GlcNAc)_3_ or (GlcNAc)_4_ was used instead of (GlcNAc)_2_, the entropy gain was even greater but this was compensated by the loss of enthalpy, resulting in lower binding affinities (Δ*G*°) of − 7.54 and − 8.36 kcal mol^−1^, respectively.

**Table 3:** Thermodynamic parameters obtained for the *Vc*CBP/(GlcNAc)_n_ (n = 2, 3, and 4) interactions by means of ITC.

		*N*	*K*_a_ (10^8^ M^−1^)	Δ*H*° (kcal·mol^−1^)	*− T*Δ*S*° (kcal·mol^−1^)	Δ*G*° (kcal·mol^−1^)
*Vc*CBP	(GlcNAc)_2_	0.9 ± 0.0	4.08 ± 1.03	− 2.40 ± 0.03	− 6.45 ± 0.11	− 8.85 ± 0.14
*Vc*CBP	(GlcNAc)_3_	1.1 ± 0.01	0.64 ± 0.14	2.02 ± 0.09	− 9.56 ± 0.03	− 7.54 ± 0.12
*Vc*CBP	(GlcNAc)_4_	1.0 ± 0.05	1.70 ± 0.04	5.47 ± 0.19	− 13.8 ± 0.16	− 8.36 ± 0.02
*Vc*CBP_R27A	(GlcNAc)_3_	0.7 ± 0.18	0.083 ± 0.061	0.92 ± 0.30	− 7.30 ± 0.55	− 6.38 ± 0.55

The individual ligands (1.0 mM) were titrated into the *Vc*CBP or *Vc*CBP_R27A solution (50 µM) in 20 mM Tris–HCl buffer, pH 8.0, in the sample cell (0.2028 mL). The experiments were performed at 25 °C using an iTC200 system (Microcal Northampton, MA, USA). Three repeated experiments were conducted to obtain individual sets of the thermodynamic parameters.

### Temperature dependences of thermodynamic parameters for (GlcNAc)_n_ binding

ITC profiles of (GlcNAc)_3_ titration into *Vc*CBP at various temperatures (10–35 °C) are shown in Supplementary Fig. S2. Heat absorption was observed up to 25 °C and converted to heat release at 30–35 °C. The binding stoichiometry of (GlcNAc)_3_/*Vc*CBP remained almost constant at approximately 1.0. The interactions were more exothermic at higher temperature. The temperature dependence was then examined for (GlcNAc)_2_ and (GlcNAc)_4_ at various temperatures from 5 to 35 °C, and the thermodynamic parameters obtained were plotted against individual temperatures. The results are shown in Supplementary Figs. S3A-S3C. The individual thermodynamic parameters (Δ*G*°, Δ*H*° and *-T*Δ*S*°) for the binding of (GlcNAc)_2_, (GlcNAc)_3_ and (GlcNAc)_4_ were linearly related to the temperature. Based on the slope of Δ*H*°, we calculated heat capacity changes (Δ*C*_p_°), which are listed in Table 4. Large negative values of Δ*C*_p_° were observed for the interactions of individual (GlcNAc)_n_ with *Vc*CBP, indicating significant hydrophobic interactions^35^. The highest Δ*C*_p_° (− 406 cal K^−1^ mol^−1^) was observed for the *Vc*CBP-(GlcNAc)_3_ interaction, while lower Δ*C*_p_° values were obtained for (GlcNAc)_2_ (− 291 cal K^−1^ mol ^−1^) and (GlcNAc)_4_ (− 307 cal K^−1^ mol^−1^). From the Δ*C*_p_° values thus obtained, we calculated the solvation entropy change (Δ*S*°_solv_) according to Eq. (2), and subsequently obtained the conformational entropy change (Δ*S*°_conf_) based on the Eq. (3). The results are also listed in Table 4. Both favorable Δ*S*°_solv_ and unfavorable Δ*S*°_conf_ contributed to the interaction with (GlcNAc)_n_. The favorable Δ*S*°_solv_ can be explained by the exclusion of bound water molecules upon ligand binding, while the unfavorable Δ*S*°_conf_ by the reduction in the fluctuation of the *Vc*CBP structure as seen in Fig. 5, which shows the fluctuations of RMSDs in unliganded and liganded states.

**Table 4:** Dissection of entropic contribution to the *Vc*CBP/(GlcNAc)_n_ (n = 2, 3, and 4) interactions.

Protein	Ligand	Δ*C*_p_° (cal K^−1^ mol^−1^)	Δ*S*_r_° (cal K^−1^ mol^−1^)	Δ*S*_mix_° (cal K^−1^ mol^−1^)	Δ*S*_solv_° (cal K^−1^ mol^−1^)	Δ*S*_conf_° (cal K^−1^ mol^−1^)
*Vc*CBP	(GlcNAc)_2_	− 291 ± 1	22.0 ± 0.4	− 8	81.6 ± 0.2	− 51.6 ± 0.6
*Vc*CBP	(GlcNAc)_3_	− 406 ± 6	32.6 ± 0.1	− 8	110.9 ± 1.8	− 70.3 ± 1.9
*Vc*CBP	(GlcNAc)_4_	− 307 ± 10	47.2 ± 0.6	− 8	84.0 ± 2.8	− 28.9 ± 3.3
*Vc*CBP_R27A	(GlcNAc)_3_	− 343 ± 1	25.6 ± 0.0	− 8	93.5 ± 0.2	− 59.9 ± 0.2

The individual ligands (1.0 mM) were titrated into the *Vc*CBP or *Vc*CBP_R27A solution (50 µM) in 20 mM Tris–HCl buffer, pH 8.0, in the sample cell (0.2028 mL) at various temperatures. Individual entropic contributions were obtained from Supplementary Fig. S3, and Eqs. (2) and (3).

### Binding experiments using *Vc*CBP_R27A

To define the contribution of the Arg27 side chain to (GlcNAc)_3_ binding, we conducted ITC analysis of the interaction with (GlcNAc)_3_ using *Vc*CBP_R27A. As in the case of *Vc*CBP, heat absorption was observed up to 20 °C, and converted to heat release at 25–35 °C (Supplementary Fig. S4). The thermodynamic parameters were obtained for the individual temperatures, and the values were plotted as shown in Supplementary Fig. S3D. At all temperatures tested, the binding affinities (Δ*G*°) were found to be reduced by about 1 kcal/mol in *Vc*CBP-R27A (Supplementary Fig. S3D, Table 3). Thus Arg27 participates in the binding of (GlcNAc)_3_ but its contribution is only moderate. The temperature dependence of Δ*H*° of *Vc*CBP_R27A indicated that both the solvation entropy gain and conformational entropy loss were reduced (Δ*S*^o^_solv_, 110.9 → 93.5 cal K^−1^ mol^−1^; Δ*S*^o^_conf_,  − 70.3 → − 59.9 cal K^−1^ mol^−1^), and the reduction in the total entropic contribution (− *T*Δ*S*^o^, − 9.56 → − 7.30 kcal mol^−1^) was compensated by the reduction in enthalpy loss (Δ*H*^o^, 2.02 → 0.92 kcal mol^−1^) (Tables 3 and 4).

## Discussion

### (GlcNAc)_2_ binds primarily to the Upper1/Lower interface (Site1/Site2)

As listed in Table 3, the interaction of (GlcNAc)_2_ with *Vc*CBP was entropy-driven (− *T*Δ*S*° = − 6.45 kcal mol^−1^) with a smaller enthalpy gain (Δ*H*° = − 2.40 kcal·mol^−1^). This is consistent with the data reported for *Vh*CBP (Δ*H*° = − 3.9 kcal·mol^−1^ and − *T*Δ*S*° = − 6.4 kcal mol^−1^)^28^. Since the amino acid residues involved in (GlcNAc)_2_ binding in *Vh*CBP are almost conserved in *Vc*CBP as shown in Fig. 1, the mechanism of (GlcNAc)_2_ binding in *Vc*CBP is similar to that in *Vh*CBP from the viewpoints of structure and thermodynamics. As shown in Fig. 3B and Supplementary Table S1, only the Upper1/Lower interface appears to be involved in the binding of two GlcNAc residues from the non-reducing end. Since *Vc*CBP has been regarded as being specific for (GlcNAc)_2_ translocation to the ABC-transporter^18^, (GlcNAc)_2_ binds primarily to the interface between the Upper1 and Lower domains (Site1/Site2) before translocation.

### Enthalpic Site1/Site2 and entropic Site3/Site4

From the thermal unfolding and ITC experiments, we found that the binding affinities of (GlcNAc)_n_ (n = 2, 3, and 4) were comparable with each other (Supplementary Fig. S1 and Fig. 9; Tables 2 and 3), but that favorable contributions of entropic term (− *T*Δ*S*°) to the binding affinities were enhanced in the order (GlcNAc)_2_ < (GlcNAc)_3_ < (GlcNAc)_4_ (Table 3). As the entropic contributions were enhanced, compensations were clearly found in the enthalpic terms. As seen from the crystal structures of (GlcNAc)_2_-liganded (1ZU0) and (GlcNAc)_3_-liganded *Vc*CBP (Fig. 3B), reducing-end GlcNAc of bound (GlcNAc)_3_ exists beyond the Upper1/Lower interface and that of bound (GlcNAc)_4_ may be in contact with the Upper2/Lower interface (Site3 and Site 4). From the temperature dependence of Δ*H*°, we found that favorable solvation entropy (Δ*S*°_solv_) predominated over conformational entropy changes (Δ*S*°_conf_) as listed in Table 4, suggesting that Site3 and Site4 bind a relatively large number of water molecules in the open form, and that the bound water molecules may be excluded upon (GlcNAc)_3_ or (GlcNAc)_4_ binding. We calculated the solvent-accessible surface areas (ASA) for unliganded, (GlcNAc)_2_-liganded, and (GlcNAc)_3_-liganded *Vc*CBPs based on their crystal structures^36^, and the data were presented in Supplementary Table 2. We found that the apolar solvent accessible surface area (ASA_apolar_) was reduced by 3.3 or 4.2% upon binding of (GlcNAc)_2_ or (GlcNAc)_3_, respectively. This indicated that (GlcNAc)_3_ binding to *Vc*CBP excludes more water molecules from the apolar surface than (GlcNAc)_2_ binding. This is consistent with the positive values of Δ*S*_solv_, which is greater in (GlcNAc)_3_ than in (GlcNAc)_2_ (Table 4). The thermodynamic data obtained for (GlcNAc)_3_ binding to *Vc*CBP_R27A (Tables 3 and 4) well supported the contribution of solvation entropy in (GlcNAc)_3_ binding. The favorable entropy change derived from solvation was suppressed by mutation of Arg27 to alanine, indicating that the bound water molecules were at least partly removed by this mutation. For the interactions of Site3 GlcNAc involving Arg27, it appears that the hydrogen bond is less significant but entropic contribution caused by the release of bound water molecules is more significant. Thus, we propose that Site1/Site2 corresponding to the Upper1/Lower interface and Site3/Site4 corresponding to the Upper1/Upper2/Lower interface can be respectively regarded as enthalpic- and entropic-interaction sites.

### Upper1 and Upper2 domains play different roles in (GlcNAc)_2_ translocation

In *Vc*CBP, we found three structural domains, the Upper1, Upper2 and Lower domains (Fig. 2), which correspond to domains I, II and III of cluster C SBPs reported by Chandravanshi et al.^19^. The three-domain organization appears to be significant from a functional viewpoint. One of the reasons for this significance derives from the clear distinction found in the binding thermodynamics between Site1/Site2 and Site3/Site4; although both enthalpy and entropy contributions were involved in the interaction with the former site, the entropic contribution predominates in the latter site (Tables 3 and 4). The second reason is the larger RMSD of the Upper2 domain upon superimposition of (GlcNAc)_2_-liganded and (GlcNAc)_3_-liganded *Vc*CBP structures (Fig. 2C). The larger RMSD is likely to produce the space for accommodating the additional GlcNAc unit at the interface between Upper1 and Upper2 domains. The third reason is the distinction in the molecular movements between the Upper1/Lower and Upper2/Lower interfaces. The distance Gln101-Gly432 fluctuated more in (GlcNAc)_3_-liganded than in (GlcNAc)_2_-liganded *Vc*CBP, whereas the distances, Asn203-Gly361 and Thr24-Ala391 were similar (Fig. 6). In (GlcNAc)_3_-liganded *Vc*CBP, the fluctuations were more intensive in the Upper2/Lower interface (Gln101-Gly432) than in the Upper1/Lower interface (Asn203-Gly361/Thr24-Ala391) as shown in Fig. 6B. The larger fluctuation in agreed well with the highest B-factor values in the loop immediately following η6 and in the loop between α14 and α15 (Fig. 4). A “half-open” conformation observed in Trp513-mutated *Vh*CBP^30^ may correspond to a snapshot of the largely fluctuated state. Cross-correlation heat maps of (GlcNAc)_2_-liganded and (GlcNAc)_3_-liganded *Vc*CBP (Fig. 8) also showed the independency of the Upper1 and Upper2 domains in their molecular movements; thus, it is most likely that Upper1 and Upper2 domains play different roles in the (GlcNAc)_2_ translocation process.

### Hypothetical releasing site, Site5/Site6

As shown in Fig. 9, titrations of *Vc*CBP with (GlcNAc)_5_ or (GlcNAc)_6_ did not result in any heat release/absorption; the binding affinities are too low to obtain the thermodynamic parameters for these oligosaccharides by ITC. Nevertheless, thermal unfolding data (Supplemental Fig. S1 and Table 2) revealed a significant elevation of the transition temperature of thermal unfolding (Δ*T*_m_ = 6.3 °C), suggesting a significant interaction of (GlcNAc)_5_ or (GlcNAc)_6_ with *Vc*CBP. In the interaction of (GlcNAc)_6_ with *Vc*CBP, the two GlcNAc residues of the non-reducing end interact with the interface between the Upper1/Lower (Site1/Site2) and the neighboring (GlcNAc)_2_ unit also interacts with the Upper1/Upper2/Lower interface (Site3/Site4). However, the additional (GlcNAc)_2_ unit of the reducing-end side may be repelled from the protein surface (hypothetical Site5/Site6). This situation may bring about the lower but significant Δ*T*_m_ in Table 2, accounting for the lower binding affinity of (GlcNAc)_5_ and (GlcNAc)_6_. We propose here that a specific substrate (GlcNAc)_2_ primarily binds Site1/Site2 with both enthalpy- and entropy-driven interactions, and is subsequently translocated to Site3/Site4, where the binding interaction is looser, leading to release of the sugar molecule from Site5/Site6 to a (GlcNAc)_2_-specific ABC transporter. The loosening of the interaction at Site3/Site4 may be caused by the higher mobility of the Upper2/Lower interface observed in the (GlcNAc)_3_-liganded structure (Figs. 4, 6 and 7). Thus, all translocation processes are conducted by the cooperative action of the three domains, Upper1, Upper2 and Lower.

### Structure triggering the (GlcNAc)_2_ unit translocation

Kitaoku et al.^30^ observed electron density of the Phe411 side chain with a full occupancy (1.0) close to the *N*-acetyl methyl group of the Site2 GlcNAc in (GlcNAc)_2_-liganded *Vh*CBP. However, in (GlcNAc)_3_-liganded *Vh*CBP, the same side chain was found 3.5 Å away from the Site2 GlcNAc with a full occupancy (1.0). Here, in (GlcNAc)_3_-liganded *Vc*CBP we observed the side chain of the corresponding phenylalanine residue (Phe410) at both positions with individual occupancies of 0.5 (Fig. 3A), indicating a significant flipping of the Phe410 side chain. The density of the Site3 GlcNAc was also observed with an occupancy of 0.5, indicating the higher mobility of the Site3 GlcNAc (Figs. 3A and 4D), which appeared to be coordinated with that of the Phe410 side chain. The coordinated motions of the phenylalanyl side chain and the Site3 GlcNAc suggested that Phe410 may be involved in the translocation of bound (GlcNAc)_2_ from Site1/Site2 to Site3/Site4. Perhaps the translocation process is triggered by interaction with the corresponding ABC transporter, which may further translocate (GlcNAc)_2_ to the hypothetical release site, Site5/Site6, located in the Upper2/Lower interface.

## Conclusion

Taken together, the three domains, Upper1, Upper2, and Lower domains, found in the crystal structure of *Vc*CBP play different roles and function cooperatively in translocation of (GlcNAc)_2_. The mechanism proposed here was fully supported by binding data obtained by thermal unfolding and ITC experiments and may be applicable to other translocation systems involving SBPs belonging to the same cluster.

## Materials and methods

### Materials

(GlcNAc)_n_ (n = 2–6) oligosaccharides were produced by acid hydrolysis of chitin^37^ and purified by gel filtration on Cellufine Gcl-25 m (JNC Co., Tokyo, 3.5 × 180 cm). Ni–NTA Agarose was purchased from QIAGEN (Tokyo, Japan). Q-Sepharose Fast Flow and HiPrep 16/60 Sephacryl S-100 were from GE Healthcare (Tokyo, Japan) and TOYOPEARL Butyl-650 M was from Tosoh (Tokyo, Japan). Other reagents were of analytical grade and commercially available.

### Construction of expression plasmid for *Vc*CBP and *Vc*CBP_R27A

Synthetic genes encoding *Vc*CBP and *Vc*CBP_R27A, in which Arg27 was mutated to alanine, were obtained from Invitrogen (Carlsabad, CA, USA). The nucleotide sequences of the genes were optimized to increase expression in *E. coli* without changing the amino acid sequences of these proteins. The expression vectors, pRham-*Vc*CBP and pRham-*Vc*CBP_R27A, were constructed by the Expresso^(R)^ Rhamnose Cloning and Expression System, N-His (Lucigen, UK).

### Protein expression and purification

The expression vector, pRham-*Vc*CBP or pRham-*Vc*CBP_R27A was transformed into *E. coli* C43(DE3). Induction with 0.2% α-L( +)-rhamnose was conducted according to the supplier’s instruction. After induction, the culture was incubated at 15 °C for 40 h, then the cells were harvested and disrupted by sonication in 20 mM Tris–HCl buffer, pH 8.0. The sonicated extract was centrifuged at 12,000×*g* for 15 min at 4 °C. The soluble fraction was dialyzed against 20 mM Tris–HCl buffer pH 8.0, and applied to a Ni–NTA column equilibrated with the same buffer. After washing the column with the Tris buffer, the bound protein fractions were eluted with a linear gradient of 0–0.2 M imidazole. The fractions containing a protein of molecular mass 60 kDa, which corresponds to that of *Vc*CBP, were collected, and ammonium sulfate was added to the protein solution to a final concentration of 1 M. The solution was applied to the TOYOPEARL Butyl 650 M column equilibrated with 20 mM Tris–HCl buffer pH 8.0 containing 1 M ammonium sulfate. The adsorbed fraction was eluted with a linear gradient of 1–0 M ammonium sulfate in 20 mM Tris–HCl buffer pH 8.0. The protein fractions containing *Vc*CBP were pooled and applied to a Q-Sepharose column previously equilibrated with the same buffer. The protein was eluted stepwise with 0.15 M NaCl in the same buffer. The fractions containing *Vc*CBP were pooled and further applied to a Sephacryl S-100 HR gel-filtration column equilibrated with the same buffer containing 0.1 M NaCl. Fractions exhibiting a single protein band on SDS-PAGE^38^ (Supplementary Fig. S5) were pooled and stored at 4 °C.

### Protein concentration

Protein concentrations were determined by reading absorbance at 280 nm, using an extinction coefficient of *Vc*CBP (110,365 M^−1^ cm^−1^) calculated from the equation proposed by Pace et al.^39^.

### Thermal unfolding experiments

To obtain the thermal unfolding curve of *Vc*CBP, the CD value at 222 nm was monitored using a Jasco J-720 spectropolarimeter (cell length 0.1 cm), while the solution temperature was raised at a rate of 1 °C min^−1^ using a temperature controller (PTC-423L, Jasco). To facilitate comparison of unfolding curves, the experimental data (molar ellipticities) were normalized to obtain unfolded fractions at individual temperatures. To assess the binding ability of GlcNAc and (GlcNAc)_n_ (n = 2, 3, 4, 5, and 6), the unfolding experiments of *Vc*CBP were conducted in the presence or absence of (GlcNAc)_n_. Individual unfolding experiments were repeated twice under the same conditions. The transition temperature of thermal unfolding (*T*_m_) was elevated when the ligand was added to the *Vc*CBP solution. The elevation of *T*_m_ (Δ*T*_m_) indicated the binding of the ligand^40^. The solvent condition was 20 mM Tris–HCl buffer pH 8.0. Final concentrations of the protein and (GlcNAc)_n_ were 8 µM and 8 mM, respectively.

### Isothermal titration calorimetry (ITC)

Solutions of 50 μM *Vc*CBP or *Vc*CBP_R27A in 20 mM Tris–HCl buffer, pH 8.0, were degassed and loaded into the sample cell (0.2028 mL). The individual ligands (1.0 mM) were dissolved in the same buffer, degassed and loaded into a syringe. Calorimetric titration was performed at 25 °C using an iTC200 system (Microcal Northampton, MA, USA). In the titrations, 2.5 μL of a ligand was injected into the sample cell at 180-s intervals with a stirring speed of 1000 rpm. The dilution heat caused by each titration was measured by titrating ligand to buffer solution without protein under identical conditions. The dilution heat thus obtained was subtracted from the heat change that was observed in the presence of protein. Individual titration experiments were repeated three times to obtain reliable values of thermodynamic parameters. The Origin software installed in the ITC instrument was used to analyze the ITC data. Using the One-set of Sites model, individual datasets obtained from the titration experiments fitted well to the theoretical curves, providing the stoichiometries (*n*), equilibrium association constants (*K*_a_) and enthalpy changes (Δ*H*°) of the protein–ligand interactions. The binding free energy change (Δ*G*°) and entropy change (Δ*S*°) were calculated from the relationship as follows,

1 $$\Delta G^\circ \, = \, - RT\cdot{\text{ ln}}K_{{\text{a}}} = \, \Delta H^\circ - T\Delta S^\circ$$

The accuracy of the thermodynamic parameters obtained was assessed from the *c*-values calculated from the equation *c* = *n*·*K*_a_·[M]_*t*_, where [M]_*t*_ is the total concentration of protein^41^. The ITC measurements were conducted in 20 mM Tris–HCl buffer pH 8.0, at various temperatures from 5 to 35 °C. The Δ*H*° values obtained for various temperatures were plotted against temperatures, and the slope of a straight line fitted to the experimental points corresponds to the heat capacity change (Δ*C*_p_°). As the entropy of solvation is regarded as zero for proteins near 385 K, Δ*C*_p_° was converted to the solvation entropy change (Δ*S*_solv_°) at 25 °C (298 K) according to the following relationship,

2 $$\Delta S\mathrm{solv}^\circ =\Delta C\mathrm{p}^\circ \mathrm{ ln}(\frac{298.15\mathrm{ K}}{385.15\mathrm{ K }})$$

The conformational entropy change (Δ*S*_conf_°) was calculated from Δ*S*° obtained from Eq. (1), the solvation entropy change (Δ*S*_solv_°) and the mixing entropy change (Δ*S*_mix_°, − 8 cal K^−1^ mol^−1^), based on the following Eq. ^42^,

3 $$\Delta S^\circ = \Delta S_{{{\text{solv}}}}^\circ + \Delta S_{{{\text{mix}}}}^\circ + \Delta S_{{{\text{conf}}}}^\circ$$

### Crystallization and data collection

Crystallization conditions for *Vc*CBP were screened using the sparse-matrix sampling method by sitting drop vapor diffusion at 20 °C. Under optimized crystallization conditions, 1 μL of protein solution (5 mg/ml in water) was mixed with 1 µL of 0.1 M sodium citrate containing 0.2 M ammonium acetate with 30% w/v polyethylene glycol 4000, pH 5.6. Rod-like crystals of *Vc*CBP grew within 3 weeks to a size of up to 0.1 × 0.1 × 0.5 mm^3^. To prepare the crystals of (GlcNAc)_3_-liganded *Vc*CBP, the unliganded crystals were transferred to the crystallization well solution containing 26 mM (GlcNAc)_3_ and incubated at 20˚C for 3.5 h. The crystals were successfully grown in the presence of (GlcNAc)_3_. For data collection, the crystals were transferred into the cryoprotectant solution containing 0.01 M zinc sulfate, 0.1 M MES (pH 6.5) and 30% PEG MME550, and then flash-cooled in a nitrogen stream at 95 K. The diffraction data were collected at the beam-line BL-17A of Photon Factory (Ibaraki, Japan), using an EIGER X 16 M (Dectris), at a cryogenic temperature (95 K). The data were integrated and scaled with XDS^43^. The processing statistics are summarized in Table 1.

### Structural determination and refinement

The structures of unliganded and (GlcNAc)_3_-liganded *Vc*CBP were solved by the molecular replacement method using the program PHASER^44^, where the structures of unliganded *Vc*CBP (PDB code, 1ZTY) and (GlcNAc)_2_-liganded *Vh*CBP (PDB code, 5YQW) served as search models, respectively. For unliganded *Vc*CBP, two protein molecules were located in the crystallographic asymmetric unit. The model was improved by several rounds of refinement with PHENIX^45^ and COOT programs^46^. Occupancies of the Site3 GlcNAc and two conformers of the Phe410 side chain were set at 0.5. The structure of unliganded *Vc*CBP was refined to an *R*_work_/*R*_free_ of 16.2/19.1% at a resolution of 1.6 Å. The final model contains two protein molecules that include residues 1–532 for each molecule and 773 water molecules. The stereochemistry of the model was verified using MolProbity^47^, showing 96.7%, 3.3% and 0% of protein residues in the favored, allowed and disallowed regions of the Ramachandran plot, respectively. For (GlcNAc)_3_-liganded *Vc*CBP, one protein molecule was located in the crystallographic asymmetric unit. The structure of (GlcNAc)_3_-liganded *Vc*CBP was refined to an *R*_work_/*R*_free_ of 16.9/18.7% at a resolution of 1.22 Å. The final model contained one protein molecule that includes residues 1–532 and 563 water molecules. The stereochemistry verification showed 97.3%, 2.7% and 0% of protein residues in the corresponding regions, respectively. Molecular graphics were illustrated with PyMol software (http://www.pymol.org/). The refinement statistics are summarized in Table 1.

### Molecular dynamics simulation

Molecular dynamics simulations were started from three different conformations of *Vc*CBP, unliganded, (GlcNAc)_2_-liganded, and (GlcNAc)_3_-liganded *Vc*CBP (PDB codes: 8I5J, 1ZU0, and 8I5K, respectively). Molecular dynamics package Gromacs^48^, version 2019 and 2020.7, were used for the simulations and analysis of the simulated data. Protein topologies were generated with Amber99SB force field^49^. The ligand topologies were generated with ACPYPE server^50^. Amber GAFF force field was used for generation of the parameters and partial charges with AM1-BCC model to correspond to HF/6-31G* RESP charges^51^. The structures were placed in a rectangular box 1 nm from the protein and solvated in a pre-equilibrated water configuration with TIP3 water model^52^. Counter ions were added to neutralize the system. The structure was energy-minimized with steepest descent algorithm. To equilibrate the system into constant temperature, pressure and density 100 ps NVT and NPT simulations constraining the heavy atoms and 100 ps NPT simulation without constraints were performed before the actual NPT production runs. Berendsen pressure coupling was used in the constrained NPT simulation^53^. In the production runs modified Berendsen method, velocity rescaling^54^ was used for temperature coupling and Parinnello and Rahman^55^ for pressure coupling. Production runs were 500 ns with time step of 2 fs and the conformations and energies were collected every 10 ps. RMSDs of the whole protein and the domains from the starting conformation of each simulation were calculated based on the backbone atoms. Principal component analysis (PCA) or essential dynamics^56^ based on the variations of Cα atom positions were used to extract the largest motions in each simulation. The cross-correlation data were obtained through ProDy server^57^ via interface from VMD^58^.

### Solvent accessibility surface area (ASA)

ASA was calculated using the method reported by Fraczkiewicz and Braun^36^ (GetArea 1.1, TX, United States), based on the crystal structures of unliganded, (GlcNAc)_2_-liganded, and (GlcNAc)_3_-liganded *Vc*CBPs.

### Supplementary Information

Supplementary Information.

## Data Availability

The atomic co-ordinates and structural factor data of *Vc*CBP have been deposited in the PDB database under the accession codes 8I5J and 8I5K for the unliganded *Vc*CBP and (GlcNAc)_3_-liganded *Vc*CBP, respectively.

## Abbreviations

SBP: Periplasmic solute-binding protein
CBP: SBP specific to chitooligosaccharide
*Vc*CBP: CBP from *Vibrio cholerae*
*Vh*CBP: CBP from *Vibrio **campbellii* (formerly, *Vibrio **harveyi*)
*Tm*CBP: SBP specific to cellobiose from *Thermotoga** maritima*
GlcNAc: *N*-Acetylglucosamine
(GlcNAc)_n_: β-1,4-Linked oligosaccharide of GlcNAc
CD: Circular dichroism
ITC: Isothermal titration calorimetry
RMSD: Root mean square deviations
ASA: Solvent-accessible surface area

## References

[1] Gooday GW (1999). Aggressive and defensive roles for chitinases. EXS.

[2] Zhang R, Zhou J, Song Z, Huang Z (2018). Enzymatic properties of β-*N*-acetylglucosaminidases. Appl. Microbiol. Biotechnol..

[3] Bonin M, Sreekumar S, Cord-Landwehr S, Moerschbacher BM (2020). Preparation of defined chitosan oligosaccharides using chitin deacetylases. Int. J. Mol. Sci..

[4] Loose JS, Forsberg Z, Fraaije MW, Eijsink VG, Vaaje-Kolstad G (2014). A rapid quantitative activity assay shows that the *Vibrio cholerae* colonization factor GbpA is an active lytic polysaccharide monooxygenase. FEBS Lett..

[5] Muthukrishnan S, Merzendorfer H, Arakane Y, Yang Q (2019). Chitin organizing and modifying enzymes and proteins involved in remodeling of the insect cuticle. Adv. Exp. Med. Biol..

[6] Nazari B, Kobayashi M, Saito A, Hassaninasab A, Miyashita K, Fujii T (2013). Chitin-induced gene expression in secondary metabolic pathways of *Streptomyces **coelicolor* A3(2) grown in soil. Appl. Environ. Microbiol..

[7] Zhang X, Lin H, Wang X, Austin B (2018). Significance of *Vibrio* species in the marine organic carbon cycle: A review. Sci. China Earth Sci..

[8] Bassler BL, Yu C, Lee YC, Roseman S (1991). Chitin utilization by marine bacteria: Degradation and catabolism of chitin oligosaccharides by *Vibrio **furnissii*. J. Biol. Chem..

[9] Suginta W, Robertson PAW, Austin B, Fry SC, Fothergill-Gilmore LA (2000). Chitinases from *Vibrio*: Activity screening and purification of chiA from *Vibrio **carchariae*. J. Appl. Microbiol..

[10] Suginta W, Vongsuwan A, Songsiriritthigul C, Prinz H, Estibeiro P, Duncan RR, Svasti J, Fothergill-Gilmore LA (2004). An endochitinase A from *Vibrio **carchariae*: Cloning, expression, mass and sequence analyses, and chitin hydrolysis. Arch. Biochem. Biophys..

[11] Keyhani NO, Li XB, Roseman S (2000). Chitin catabolism in the marine bacterium *Vibrio **furnissii*. Identification and molecular cloning of a chitoporin. J. Biol. Chem..

[12] Suginta W, Chumjan W, Mahendran KR, Janning P, Schulte A, Winterhalter M (2013). Molecular uptake of chitooligosaccharides through chitoporin from the marine bacterium *Vibrio **harveyi*. PLoS ONE..

[13] Suginta W, Chumjan W, Mahendran KR, Schulte A, Winterhalter M (2013). Chitoporin from *Vibrio **harveyi*, a channel with exceptional sugar specificity. J. Biol. Chem..

[14] Aunkham A, Zahn M, Kesireddy A, Pothula KR, Schulte A, Baslé A, Kleinekathöfer U, Suginta W, van den Berg B (2018). Structural basis for chitin acquisition by marine *Vibrio* species. Nat. Commun..

[15] Keyhani NO, Roseman S (1996). The chitin catabolic cascade in the marine bacterium *Vibrio **furnissii*. Molecular cloning, isolation, and characterization of a periplasmic chitodextrinase. J. Biol. Chem..

[16] Keyhani NO, Roseman S (1996). The chitin catabolic cascade in the marine bacterium *Vibrio **furnissii*. Molecular cloning, isolation, and characterization of a periplasmic b-*N*-acetylglucosaminidase. J. Biol. Chem..

[17] Suginta W, Chuenark D, Mizuhara M, Fukamizo T (2010). Novel β-*N*-acetylglucosaminidases from *Vibrio **harveyi* 650: Cloning, expression, enzymatic properties, and subsite identification. BMC Biochem..

[18] Keyhani NO, Wang LX, Lee YC, Roseman S (1996). The chitin catabolic cascade in the marine bacterium *Vibrio **furnissii*. Characterization of an *N, *N'-diacetyl chitobiose transport system. J. Biol. Chem..

[19] Chandravanshi M, Tripathi SK, Kanaujia SP (2021). An updated classification and mechanistic insights into ligand binding of the substrate-binding proteins. FEBS Lett..

[20] Ortega Á, Matilla MA, Krell T (2022). The repertoire of solute-binding proteins of model bacteria reveals large differences in number, type, and ligand range. Microbiol. Spectr..

[21] Li X, Roseman S (2004). The chitinolytic cascade in *Vibrios* is regulated by chitin oligosaccharides and a two-component chitin catabolic sensor/kinase. Proc. Natl. Acad. Sci. USA..

[22] Klancher CA, Yamamoto S, Dalia TN, Dalia AB (2020). ChiS is a noncanonical DNA-binding hybrid sensor kinase that directly regulates the chitin utilization program in *Vibrio cholerae*. Proc. Natl. Acad. Sci. USA..

[23] Scheepers GH, Lycklama JA, Poolman B (2016). An updated structural classification of substrate-binding proteins. FEBS Lett..

[24] Fukamizo T, Kitaoku Y, Suginta W (2019). Periplasmic solute-binding proteins: Structure classification and chitooligosaccharide recognition. Int. J. Biol. Macromol..

[25] Shears P (1994). Cholera. Ann. Trop. Med. Parasitol..

[26] Austin B, Zhang XH (2006). *Vibrio **harveyi*: A significant pathogen of marine vertebrates and invertebrates. Lett. Appl. Microbiol..

[27] Chekan JR, Kwon IH, Agarwal V, Dodd D, Revindran V, Mackie RI, Cann I, Nair SK (2014). Structural and biochemical basis for mannan utilization by Caldanaerobius polysaccharolyticus strain ATCC BAA-17. J. Biol. Chem..

[28] Cuneo MJ, Beese LS, Hellinga HW (2009). Structural analysis of semi-specific oligosaccharide recognition by a cellulose-binding protein of *Thermotoga** maritima* reveals adaptations for functional diversification of the oligopeptide periplasmic binding protein fold. J. Biol. Chem..

[29] Suginta W, Sritho N, Ranok A, Bulmer DM, Kitaoku Y, van den Berg B, Fukamizo T (2018). Structure and function of a novel periplasmic chitooligosaccharide-binding protein from marine *Vibrio* bacteria. J. Biol. Chem..

[30] Kitaoku Y, Fukamizo T, Kumsaoad S, Ubonbal P, Robinson RC, Suginta W (2021). A structural model for (GlcNAc)_2_ translocation via a periplasmic chitooligosaccharide-binding protein from marine *Vibrio* bacteria. J. Biol. Chem..

[31] Chaudhuri BN, Ko J, Park C, Jones TA, Mowbray SL (1999). Structure of D-allose binding protein from *Escherichia coli* bound to D-allose at 1.8 A resolution. J. Mol. Biol..

[32] Borrok MJ, Kiessling LL, Forest KT (2007). Conformational changes of glucose/galactose-binding protein illuminated by open, unliganded, and ultra-high-resolution ligand-bound structures. Protein Sci..

[33] Anamizu K, Takase R, Hio M, Watanabe D, Mikami B, Hashimoto W (2022). Substrate size-dependent conformational changes of bacterial pectin-binding protein crucial for chemotaxis and assimilation. Sci. Rep..

[34] Pace CN, McGrath T (1980). Substrate stabilization of lysozyme to thermal and guanidine hydrochloride denaturation. J. Biol. Chem..

[35] Zolotnitsky G, Cogan U, Adir N, Solomon V, Shoham G, Shoham Y (2004). Mapping glycoside hydrolase substrate subsites by isothermal titration calorimetry. Proc. Natl. Acad. Sci. USA..

[36] Fraczkiewicz R, Braun W (1998). Exact and efficient analytical calculation of the accessible surface areas and their gradients for macromolecules. J. Comput. Chem..

[37] Rupley JA (1964). The hydrolysis of chitin by concentrated hydrochloric acid, and the preparation of low-molecular weight substrates for lysozyme. Biochim. Biophys. Acta.

[38] Laemmli UK (1970). Cleavage of structural proteins during the assembly of the head of bacteriophage T4. Nature.

[39] Pace CN, Vajdos F, Fee L, Grimsley G, Gray T (1995). How to measure and predict the molar absorption coefficient of a protein. Protein Sci..

[40] Honda Y, Fukamizo T, Boucher I, Brzezinski R (1997). Substrate binding to the inactive mutants of *Streptomyces* sp. N174 chitosanase: indirect evaluation from the thermal unfolding experiments. FEBS Lett..

[41] Turnbull WB, Daranas AH (2003). On the value of c: Can low affinity systems be studied by isothermal titration calorimetry?. J. Am. Chem. Soc..

[42] Ohnuma T, Sørlie M, Fukuda T, Kawamoto N, Taira T, Fukamizo T (2011). Chitin oligosaccharide binding to a family GH19 chitinase from the moss *Bryum **coronatum*. FEBS J..

[43] Kabsch W (2010). XDS. Acta Crystallogr. D.

[44] McCoy AJ, Grosse-Kunstleve RW, Adams PD, Winn MD, Storoni LC, Read RJ (2007). Phaser crystallographic software. J. Appl. Crystallogr..

[45] Afonine PV, Grosse-Kunstleve RW, Echols N, Headd JJ, Moriarty NW, Mustyakimov M, Terwilliger TC, Urzhumtsev A, Zwart PH, Adams PD (2012). Towards automated crystallographic structure refinement with phenix.refine. Acta Crystallogr. D..

[46] Emsley P, Cowtan K (2004). Coot: Model-building tools for molecular graphics. Acta Crystallogr. D..

[47] Williams CJ, Headd JJ, Moriarty NW, Prisant MG, Videau LL, Deis LN, Verma V, Keedy DA, Hintze BJ, Chen VB, Jain S, Lewis SM, Arendall WB, Snoeyink J, Adams PD, Lovell SC, Richardson JS, Richardson DC (2018). MolProbity: More and better reference data for improved all-atom structure validation. Protein Sci..

[48] van Der Spoel D, Lindahl E, Hess B, Groenhof G, Mark AE, Berendsen HJC (2005). GROMACS: Fast, flexible, and free. J. Comput. Chem..

[49] Lindorff-Larsen K, Piana S, Palmo K, Maragakis P, Klepeis JL, Dror RO, Shaw DE (2010). Improved side-chain torsion potentials for the Amber ff99SB protein force field. Proteins.

[50] Sousa Da Silva AW, Vrancen WF (2012). ACPYPE: AnteChamber PYthon Parser interfacE. BMC Res. Not..

[51] Jakalian A, Jack DB, Bayly CI (2002). Fast, efficient generation of high-quality atomic charges. AM1-BCC model: II Parameterization and validation. J. Comput. Chem..

[52] Jorgensen WL, Chandrasekhar J, Madura JD, Impey RW, Klein ML (1983). Comparison of simple potential functions for simulating liquid water. J. Chem. Phys..

[53] Berendsen HJC, Postma JPM, DiNola A, Haak JR (1984). Molecular dynamics with coupling to an external bath. J. Chem. Phys..

[54] Bussi G, Donadio D, Parrinello M (2007). Canonical sampling through velocity rescaling. J. Chem. Phys..

[55] Parrinello M, Rahman A (1981). Polymorphic transitions in single crystals: A new molecular dynamics method. J. Appl. Phys..

[56] Amadei A, Linssen ABM, Berendsen HJC (1993). Essential dynamics of proteins. Proteins..

[57] Bakan A, Meireles LM, Bahar I (2011). ProDy: Protein dynamics inferred from theory and experiments. Bioinformatics.

[58] Humphrey W, Dalke A, Schulten K (1996). VMD: Visual molecular dynamics. J. Mol. Graph..

