# Supplementary material for: Periplasmic chitooligosaccharide-binding protein requires a three-domain organization for substrate translocation
Source: Sci Rep. 2023 Nov 23;13:20558. doi: 10.1038/s41598-023-47253-y (PMC10667598; doi:10.1038/s41598-023-47253-y)
Supplement: Supplementary file 1 — Supplementary Information. [file 41598_2023_47253_MOESM1_ESM.docx]

**Supplementary Information**

**Periplasmic chitooligosaccharide-binding protein requires a three-domain organization for substrate translocation**

Takayuki Ohnuma^1,2,^*, Jun Tsujii^1^, Chikara Kataoka^1^, Teruki Yoshimoto^1^, Daijiro Takeshita^3^, Outi Lampela^4^, André H. Juffer^4^, Wipa Suginta^5^, & Tamo Fukamizo^1,^*

^1^Department of Advanced Bioscience, Kindai University, 3327-204 Nakamachi, Nara, 631-8505 Japan,

^2^Agricultural Technology and Innovation Research Institute (ATIRI), Kindai University, 3327-204, Nakamachi, Nara, 631-8505, Japan

^3^Biomedical Research Institute, National Institute of Advanced Industrial Science and Technology (AIST), 1-1-1 Higashi, Tsukuba-shi, Ibaraki 305-8566, Japan,

^4^Biocenter Oulu and Faculty of Biochemistry and Molecular Medicine, University of Oulu, P.O.Box 5000, FI-90014, Finland, and

^5^School of Biomolecular science & engineering, Vidyasirimedhi Institute of Science and Technology (VISTEC). Wangchan Valley 555 Moo 1 Payupnai, Wangchan, Rayong 21210 Thailand

**Supplemental figures and tables with legends**

**Figure S1.** Thermal unfolding experiments

**Figure S2.** ITC profiles of (GlcNAc)_3_ binding to *Vc*CBP

**Figure S3.** Temperature dependence of the thermodynamic parameters of (GlcNAc)_3_ binding to

*Vc*CBP

**Figure S4.** ITC profiles of (GlcNAc)_3_ binding to *Vc*CBP_R27A

**Figure S5.** SDS-PAGE profiles for VcCBP and R27A_*Vc*CBP

**Table S1.** Amino acid residues interacting with the ligand in the (GlcNAc)_3_-liganded *Vc*CBP.

**Table S2.** Solvent accessible surface areas of unliganded, (GlcNAc)_2_-liganded, and (GlcNAc)_3_-

liganded *Vc*CBPs.


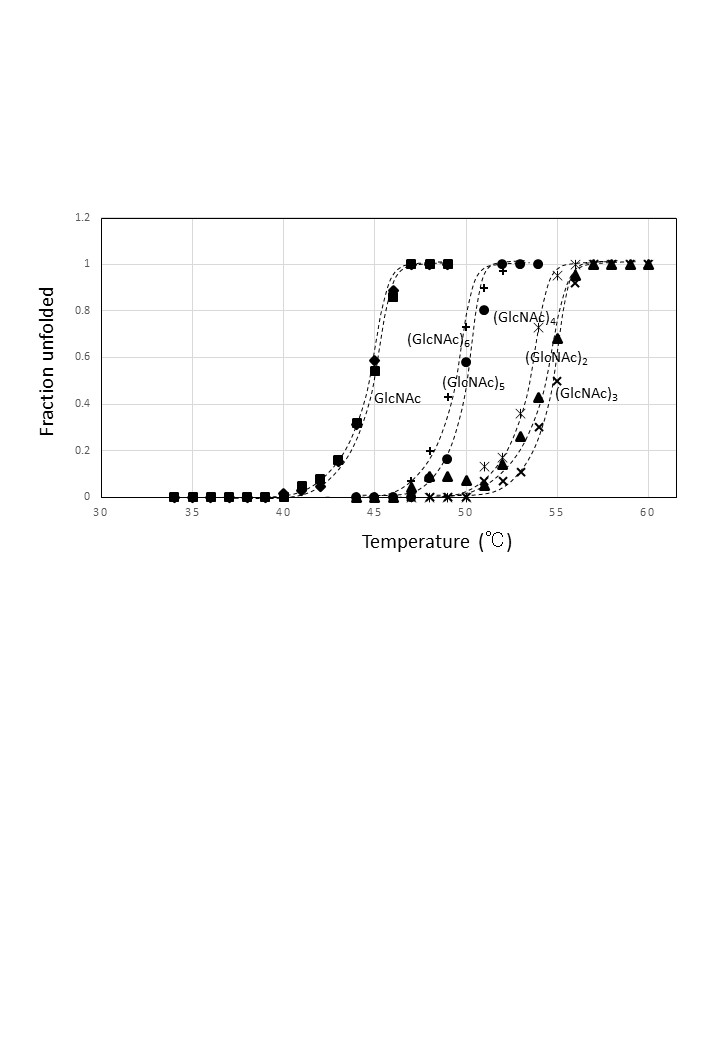


**Supplementary Figure S1. Thermal unfolding curves of *Vc*CBP in the absence or presence of (GlcNAc)_n_ (n = 2, 3, 4, 5 or 6).** The unfolding experiments were conducted in 20 mM Tris-HCl buffer pH 8.0. Final concentrations of the protein and (GlcNAc)_n_ were 8 μM and 8 mM, respectively. The unfolded fractions were obtained by monitoring CD at 220 nm. closed diamond, unliganded protein; closed square, GlcNAc; closed triangle, (GlcNAc)_2_; cross (x), (GlcNAc)_3_; asterisk (*), (GlcNAc)_4_; closed circle, (GlcNAc)_5_; plus (+), (GlcNAc)_6_.


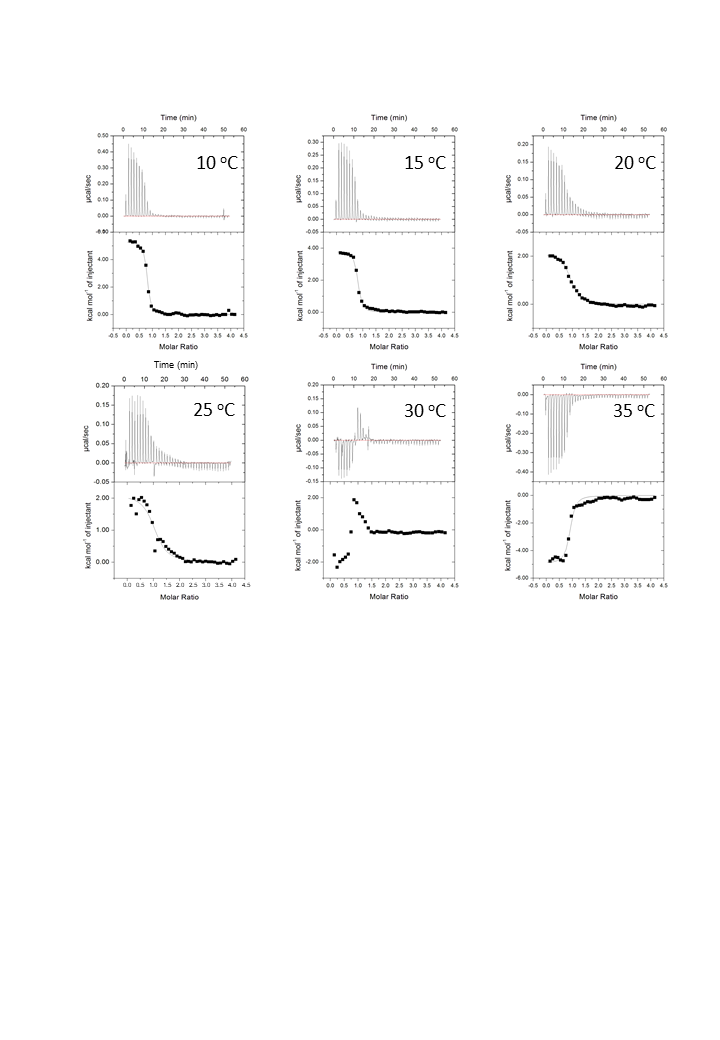


**Supplementary Figure S2. ITC analysis of the interaction of *Vc*CBP with (GlcNAc)_3_ at various temperatures.** Buffer, 20 mM Tris-HCl buffer, pH 8.0. Protein concentration was 50 μM.


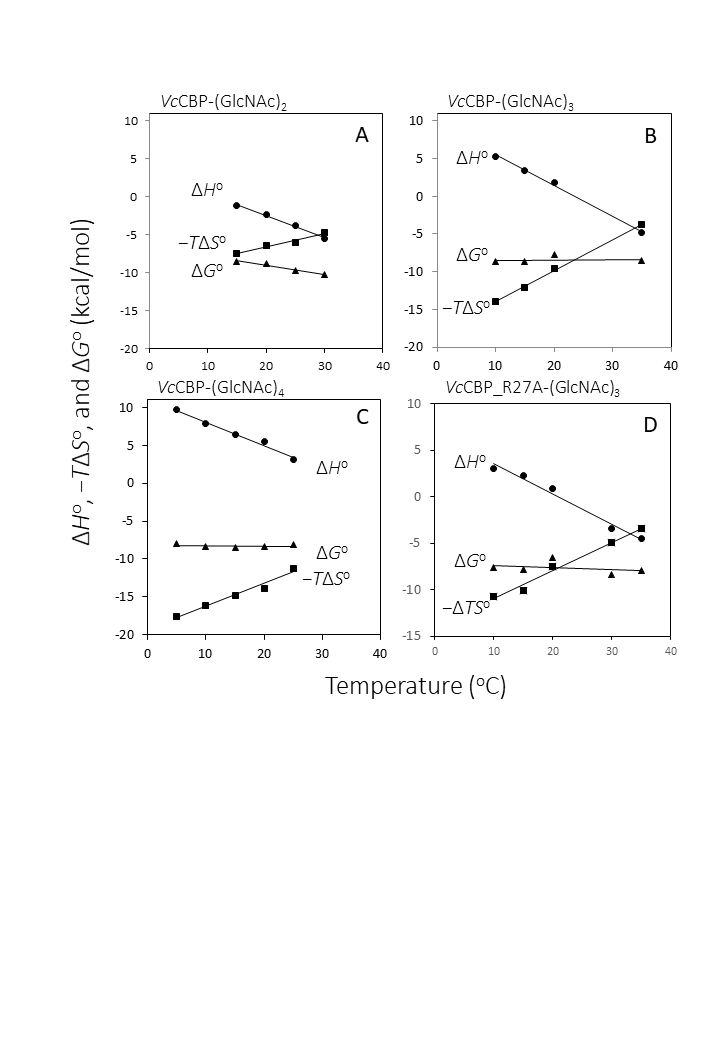


**Supplementary Figure S3.** **Temperature-dependence of the thermodynamic parameters for (GlcNAc)_n_ binding.** The data were obtained from titrations of *Vc*CBP with (GlcNAc)_2_ (**A**), (GlcNAc)_3_ (**B**) or (GlcNAc)_4_ (**C**) and from titration of *Vc*CBP_R27A with (GlcNAc)_3_ (**D**). The other experimental conditions were the same as in Figure 9.


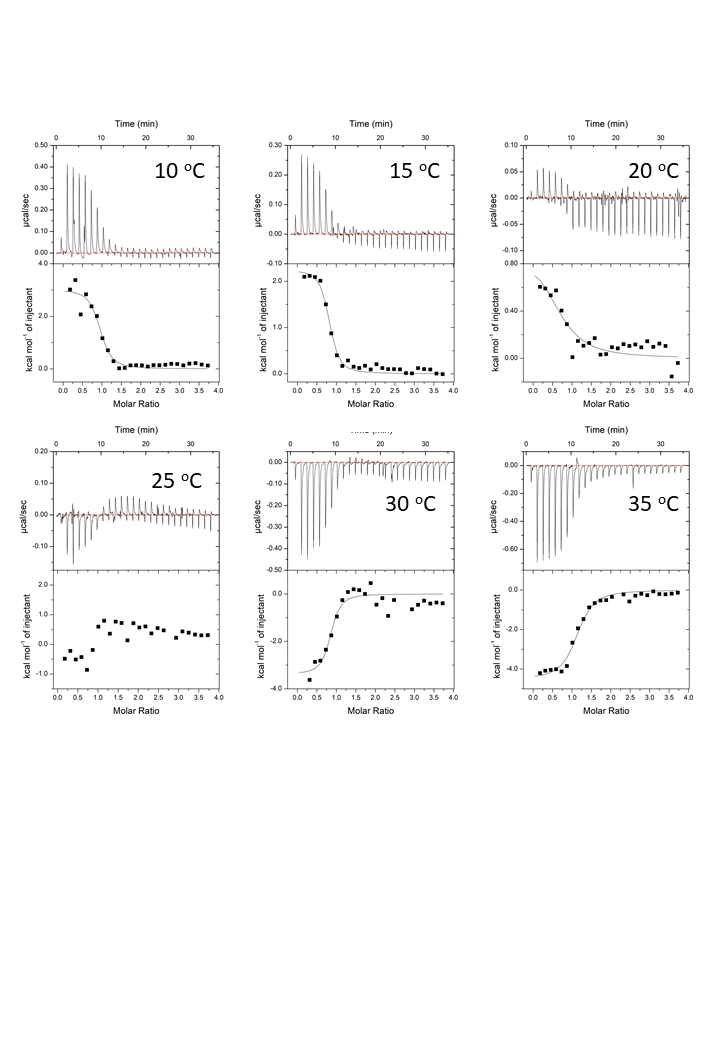


**Supplementary Figure S4. ITC analysis of the interaction of *Vc*CBP_R27A with (GlcNAc)_3_ at various temperatures.** Buffer, 20 mM Tris-HCl buffer, pH 8.0. Protein concentration was 50 μM.


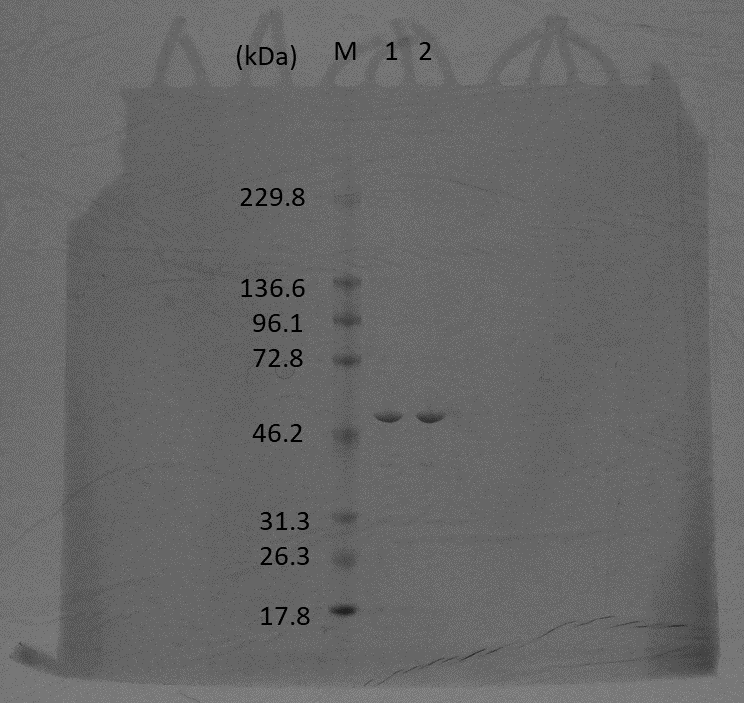


**Supplementary Figure S5. SDS-PAGE of purified *Vc*CBP and *Vc*CBP_R27A.**

M : DynaMarker Protein MultiColor Stable (BioDynamics Laboratory Inc., Tokyo, Japan) (Myosin 229.8 kDa, *β*-Galactosidase 136.6 kDa, Phosphorylase-b 96.1 kDa, BSA 72.8 kDa, Ovalbumin 46.2 kDa, Carbonic anhydrase 31.3 kDa, Tripsin inhibitor 26.3 kDa, Lysozyme 17.8 kDa). Lane 1 : purified *Vc*CBP. 2 : purified *Vc*CBP_R27A.

**Supplementary Table S1. Amino acid residues interacting with the ligand in the (GlcNAc)_3_-liganded *Vc*CBP.**

| Subsite | Hydrogen bonds | Hydrophobic interactions |
| --- | --- | --- |
| Site1 | Asn203, Ser220, Phe221, Asp364, Trp512 | Asp9, Phe10, Trp362, Asp364, Phe365 |
| Site2 | Asp9, Asn408, Arg435 | Asn408, Tyr409, Phe410, Trp512 |
| Site3 | Arg27, Ala513 | Arg27, Ala513 |

**Sipplementary Table S2. Solvent accessible surface areas (ASAs) of unliganded, (GlcNAc)_2_-liganded, and (GlcNAc)_3_-liganded *Vc*CBPs.**

| Structures | ASA*_apolar_*(Å^2^) | ASA*_polar_*(Å^2^) | ASA*_total_*(Å^2^) |
| --- | --- | --- | --- |
| *Vc*CBP | 12612.5 | 7974.3 | 20586.8 |
| (GlcNAc)_2-_liganded *Vc*CBP | 12200.2 | 7701.7 | 19901.9 |
| (GlcNAc)_3-_liganded *Vc*CBP | 12084.9 | 7808.9 | 19893.8 |
